# eHealth: A Survey of Architectures, Developments in mHealth, Security Concerns and Solutions

**DOI:** 10.3390/ijerph192013071

**Published:** 2022-10-11

**Authors:** Caroline Omoanatse Alenoghena, Adeiza James Onumanyi, Henry Ohiani Ohize, Achonu Oluwole Adejo, Maxwell Oligbi, Shaibu Ibrahim Ali, Supreme Ayewoh Okoh

**Affiliations:** 1 Department of Telecommunication Engineering, Federal University of Technology, Minna P.M.B. 65, Nigeria; 2 Next Generation Enterprises and Institutions, Council for Scientific and Industrial Research (CSIR), Pretoria 0001, South Africa

**Keywords:** architectures, eHealth, mobile computing, review, security

## Abstract

The ramifications of the COVID-19 pandemic have contributed in part to a recent upsurge in the study and development of eHealth systems. Although it is almost impossible to cover all aspects of eHealth in a single discussion, three critical areas have gained traction. These include the need for acceptable eHealth architectures, the development of mobile health (mHealth) technologies, and the need to address eHealth system security concerns. Existing survey articles lack a synthesis of the most recent advancements in the development of architectures, mHealth solutions, and innovative security measures, which are essential components of effective eHealth systems. Consequently, the present article aims at providing an encompassing survey of these three aspects towards the development of successful and efficient eHealth systems. Firstly, we discuss the most recent innovations in eHealth architectures, such as blockchain-, Internet of Things (IoT)-, and cloud-based architectures, focusing on their respective benefits and drawbacks while also providing an overview of how they might be implemented and used. Concerning mHealth and security, we focus on key developments in both areas while discussing other critical topics of importance for eHealth systems. We close with a discussion of the important research challenges and potential future directions as they pertain to architecture, mHealth, and security concerns. This survey gives a comprehensive overview, including the merits and limitations of several possible technologies for the development of eHealth systems. This endeavor offers researchers and developers a quick snapshot of the information necessary during the design and decision-making phases of the eHealth system development lifecycle. Furthermore, we conclude that building a unified architecture for eHealth systems would require combining several existing designs. It also points out that there are still a number of problems to be solved, so more research and investment are needed to develop and deploy functional eHealth systems.

## 1. Introduction

The impact of the coronavirus (COVID-19) pandemic has undoubtedly put a strain on global healthcare systems, ranging from capacity constraints to inadequate healthcare personnel protection [1]. As a result, there is an urgent need for the facilitation and deployment of eHealth technologies and resources as a viable approach to addressing present and future public health emergencies. eHealth will significantly reduce the pandemic’s negative impact on health care services by providing vital sign monitoring systems integrated with real time e-clinic management platforms with remote consultation and interaction through the use of information and communication technologies such as computers, the Internet, and mobile devices [2]. Existing healthcare systems are typically based on a ‘provider-driven’ system; however, with the invention of eHealth, such systems can be remodeled into a ‘patient-centric’ system that empowers patients to self-manage their health to a greater extent [2]. This will help to shift healthcare provisioning away from the institutional and hospital settings and toward patients’ homes, thus reducing healthcare cost. eHealth also serves as a repository for information sharing, ensuring better diagnosis and treatment information that can be instantly shared among patients, doctors, nurses, caregivers, and patients’ families. During treatment, information about a patient’s symptoms, treatments, and effectiveness can be quickly shared between medical institutions, ultimately assisting in the development of new effective treatments.

Many critical components, such as health informatics, electronic health records, telemedicine, mobile Health (mHealth), architectures, and security solutions, are required for the successful implementation of eHealth services [3]. To this end, the literature has a number of review articles covering a variety of key aspects of designing successful eHealth systems. Specifically, many of the current survey studies have consolidated the literature on subjects such as the growth of eHealth in various countries [4,5] and the standardization of eHealth technologies [6,7,8]. Other survey articles have focused on cloud-based, Internet of Things (IoT), and machine-to-machine (M2M) technological advancements [9,10]. Nevertheless, there are three other essential components of eHealth systems that, although seeing significant development in research, have received either minimal or no survey coverage in the existing body of scholarly work. These include the availability of viable architectures for eHealth deployment, advancements in the field of mHealth, and existing security solutions for preserving vital health information within eHealth platforms. The concept of architecture is important in eHealth, and it has been the focus of several research projects aimed at developing efficient and effective reference architectures. In this case, reference architectures are required not only to ensure standardization but also to define guidelines for deploying these architectures in eHealth. For example, by deploying a well designed architecture, eHealth applications situated at a doctor’s premises can be used to request remotely the temperature of a patient via body mounted sensors. This leads obviously to several benefits including increased unobtrusive surveillance and remote assessments, which improves patient safety and quality of life; increased autonomy and proactivity in terms of their well being and pathology; and more efficient and less expensive eHealth systems [11]. Consequently, there is a growing need to provide a detailed summary of such existing state-of-the-art architectures towards the facilitation and deployment of eHealth systems.

mHealth is also an essential component of eHealth as it comprises the use of mobile communication devices and wearable sensors for the delivery of healthcare services, and the gathering and transmission of health data [12]. It is an essential tool for collecting community and clinical health data, distributing and exchanging healthcare information with clinicians, researchers, and patients, tracking patient vital signs in real time, and teaching and working with healthcare professionals. Consequently, there is an undeniable connection between eHealth and mHealth, and it is essential to provide an overview of the most recent trends and advancements in mHealth towards ensuring the successful implementation of eHealth services. Lastly, the adoption of eHealth services cannot be assured without the protection of critical and highly personal information that is routinely transmitted via the platform. A large body of work has focused on providing security solutions for eHealth systems, and it is timely to examine these cutting-edge solutions. This will include issues such as privacy strategies, secure data collection and storage, and infrastructure management systems that use cryptographic keys.

Consequently, the purpose of this article is to provide a survey of existing architectures for deploying eHealth services, with a particular emphasis on blockchain, IoT, and cloud-based architectures. We also discuss mHealth developments and security solutions for eHealth platforms. In light of this, the contributions of the present survey can be summarized as follows:

1. We discuss the various architectures deployed in eHealth from a structural standpoint, narrowing our focus to the various areas in which these architectures are implemented in eHealth. The various architectures noted for eHealth system deployment are extensively discussed, with a focus on Blockchain, IoT, and cloud-based architectures.
2. We provide an overview of mHealth, a subset of eHealth, and its components, which include mobile computing devices, wireless sensors, and communication technologies. We also highlighted notable cutting-edge mHealth technologies in wireless communication, their bandwidth, coverage area, and mobility, as deployed in eHealth, to provide researchers with an overview of the most recent advances in this domain.
3. We highlight the special needs of patient privacy, secured data collection, transmission/retransmission, data storage, and eHealth infrastructure management, as well as future research challenges in security and privacy for eHealth record keeping. Recommendations for future improvement are also suggested.

A general outline of the rest of this article is given as follows: Section 2 provides a discussion of related survey articles as a means of distinguishing the present article from existing survey articles. Section 4 gives a broad overview of eHealth, while Section 5 highlights some of the different basic architectures in eHealth. In Section 6, we discuss mHealth and the basic components of mHealth deployed in eHealth. Security and privacy in eHealth are discussed in Section 7. Section 8 highlights the research challenges and future research directions, while the conclusion is presented in Section 9.

## 2. Related Literature Surveys

**Figure 1:**
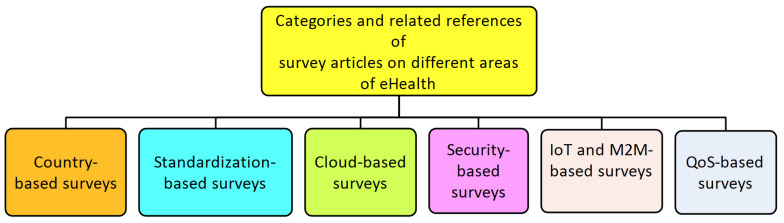
Major components of eHealth.

There is a balanced distribution of survey articles covering various aspects of eHealth service development and deployment. Our goal in this section is to provide a taxonomy of the various areas covered in the survey literature as well as to direct interested readers to the relevant sources of such information. Following our observations, these survey articles can be classified as shown in Figure 1 into the following categories: survey articles concerned with country-based deployment of eHealth, standardization issues with eHealth, cloud-, security-, IoT and machine-to-machine (M2M)-, and quality-of-service (QoS)-based survey articles.

The survey articles in [4,5] are examples of country-based survey articles that focus on issues confronting eHealth development in individual countries, with Australia and Bangladesh as case studies. Specifically, the authors of [4] provided insight into the use of the International Organization for Standardization (ISO) Open Distributed Processing (ODP) family of standards to address interoperability issues in the Australian eHealth environment. The Australian health system has a distinct institutional structure and funding model that involves the federal, state, territory, and local governments, as well as the private sector. This structure necessitates a thorough understanding of the policy environment, which includes legal, regulatory, and other enterprise policies and governance models. This presupposes the need for interoperability, which the authors define as “the ability of one system or process to use the information and/or functionality of another system or process by adhering to common standards” [4]. The authors of [5], on the other hand, investigated the current state of eHealth in the public and private sectors, as well as the technical and managerial challenges confronting eHealth projects in Bangladesh. Both articles are useful because they provide background for the need to study the potential challenges and capacity for national eHealth service implementation in various countries worldwide. Nonetheless, we highlight that neither of the papers covers every facet of eHealth, including the architectural frameworks used in its design, mHealth concerns, and security. Therefore, this offers a gap that needs deeper literature synthesis.

A few survey articles concentrated primarily on issues concerning the eHealth standardization process. In [6], for example, the authors provided a comprehensive overview of several standardization activities for M2M communications, analyzing some of the enabling technologies and applications of M2M in industry sectors such as smart grid and eHealth. Their survey is valuable since it was constructed to identify the major challenges in the design of M2M systems, the standards intended to solve these challenges, and the research concerns and directions necessary in this respect. In a different article, the authors of [7] provided insight into issues of relevant eHealth infrastructure standards, with specific reference to obstacles and limitations in radio access technology infrastructure in eHealth. Their survey also identified and analyzed the motivating factors behind future network specifications, demonstrating that it is a more accurate method of assessing future requirements than evaluating technical performance indicators of the most recent technology. In [8], the standardization of M2M communications in eHealth was also discussed. The authors discussed the key enabling IoT technologies that allow various devices (e.g., cameras, computers, sensors, smart phones, personal health devices) to communicate with one another via heterogeneous networks such as wireless local area networks (WLANs), cellular, wired Ethernet, or power line communications (PLCs). The authors of [13] also investigated the standardization of electronic health records (EHRs). They emphasized the importance of understanding the role of EHR databases, the need for standardization to improve quality, establishing interoperability in maintaining EHRs, explicating a framework for standardization and interoperability, exploring various data models for managing EHRs, and comprehending the difficulties in querying data in EHR and eHealth systems. The aforementioned survey studies have mostly focused on standardization concerns pertaining to M2M communication, design, and EHR database development. Essentially, these articles are useful for gaining an awareness of the problems involved in delivering standards, such as achieving the quality of service criteria and dependability levels required for eHealth systems. They have placed more emphasis on the role that IoT systems play in the fulfillment of functional eHealth service delivery; yet, they cannot synthesize all areas within eHealth, thus leaving gaps to explored with regards to other aspects of eHealth, such as those targeted by our current survey.

The articles in [9,10] are key examples of surveys on cloud computing in eHealth, which is an emerging paradigm in the health sector. For example, the discussion in [9] exhaustively covered topics such as the importance of cloud computing in eHealth, the challenges of cloud computing and its components, as well as existing solutions. The authors therein also discussed the need for machines to communicate with one another in the cloud. Similarly, the survey article in [14] centered on cloud concerns in eHealth. Here, the authors focused on EHR security and privacy, cloud-based eHealth data security and privacy requirements, EHR cloud architecture, and various EHR cryptographic and non-cryptographic techniques. They also raised several critical concerns, as well as the potential for further research in the field of EHR security and privacy. These specific survey studies will be beneficial to researchers who are interested in the design of cloud computing techniques for eHealth systems since they offer recommendations on how to install such systems. However, other important topics that the authors were unable to cover due to scoping factors are discussed in other relevant articles.

On the issue of security, the authors of [15] discussed the security challenges of patient health information (PHI) in eHealth and provided insights into security and privacy concerns, as well as providing a comprehensive overview of biometric technology’s role in addressing eHealth security challenges. Another survey in [16] discussed medical data security and privacy. The authors provided an overview of the challenges associated with medical data analysis and security, as well as a solution that will serve as the foundation for improving medical service quality. In parallel, the authors of [17] concentrated on the security and privacy of medical data in the Internet of Things. They looked into the use of intelligent techniques in health and how it has evolved over time, as well as the integration of IoT devices and cloud computing. Researchers who may be interested in resolving security vulnerabilities in eHealth systems, particularly as they apply to the use of IoT devices inside such systems, will find these survey articles particularly useful. The structure of these security-based survey articles also influenced our selection of the unaddressed areas within eHealth systems, such as the particular threats aimed at different infrastructures within eHealth systems and how such threats may be mitigated. Such a void must be investigated in terms of existing solutions and how they might be combined to aid future research concepts.

Other surveys provide an overview of the most recent developments in IoT and M2M devices and communications. For example, the authors of [11] investigated IoT devices in a medical context. They emphasized the broad scope of IoT-powered health care applications, as well as their speed and precision of response. Furthermore, the researchers examined an information technology architecture to ensure data security and privacy during transmission. In a different article, the authors of [18] provided an overview of the difficulties in implementing 5G technology in wireless body area network (WBAN) health care applications. They discussed how to use 5G technologies intelligently in a WBAN health care application. They also presented an overview of integrating a WBAN health care system with 5G technology, as well as the architecture of a 5G-based WBAN. Furthermore, the role of millimeter wave (mm-wave) in WBAN communication and the role of 5G in WBAN communication were discussed. Another article in [19] discussed IoT devices, with a focus on the numerous security risks associated with Bluetooth communication for eHealth systems. Here, the authors provided some examples of attacks on eHealth systems based on Bluetooth vulnerabilities. This set of review articles were able to synthesize the vast body of knowledge pertaining to communication technologies, their capacity to support eHealth systems, and the specific application domains in which they may be most applicable. Therefore, these articles will be valuable for developers who may require a comprehensive grasp of the communication requirements necessary for designing and deploying eHealth systems. Nonetheless, like with other survey articles, not every aspect of eHealth can be included; therefore, there is potential to synthesize other aspects as embarked upon in other articles, including the present survey.

Regarding QoS in eHealth, the authors of [20,21] discussed QoS in wireless eHealth, respectively, taking into account handoff schemes for QoS in wireless networking and key features of modern eHealth applications. Both survey articles focused on wireless healthcare information systems and proposed several factors to be considered for data delivery in wireless healthcare networks, such as availability, confidentiality and privacy, data delivery latency, reliability, QoS provisioning, and mobility support. The survey in [22] focused on social networks in eHealth. Here, the authors focused on social media user attitudes and knowledge extraction. They also provided an in-depth breakdown of users’ basic information, social status, and social networking experience. Some earlier survey articles focused on existing wireless technologies for deploying eHealth services, for example, in [23] where the authors discussed wireless technologies for eHealth. However, the technologies described in [23], such as the 3G networks, are now considered to be obsolete because they have been superseded by more modern technologies such as 5G networks.

Essentially, Table 1 summarizes all of these survey articles and their respective areas of focus arranged chronologically based on their year of publication. As can be seen in Table 1, the early concerns in eHealth development included QoS difficulties and the adoption of eHealth on a country-by-country basis; however, cloud, IoT, and security issues have dominated the trends in more recent times. However, when it comes to planning, developing, and implementing successful eHealth systems, there are several building blocks that must be studied beforehand. From an engineering standpoint, this will entail establishing the most appropriate architecture to be utilized, how such an architecture can be efficiently integrated with mHealth applications and services, and how such an eHealth system can be secured. Despite the existence of a significant body of literature that provides solutions to these difficulties in a variety of ways, there is a striking absence of survey articles that synthesize these studies. Consequently, this suffices as a potential gap that needs to be addressed and direct topics examined within the present article were thus constructed based on these aforementioned questions. Furthermore, Table 1 demonstrates, to the best of the authors’ knowledge, that there have been few or no broad survey papers on contemporary architectures for eHealth designs and mHealth technologies. In addition, the most recent survey article about issues regarding security was conducted in 2019, and a number of improvements have taken place since then. As a result, the purpose of this article is to provide a comprehensive review regarding trends and advancements in these three aspects of eHealth system and service delivery in order to fill the void that this knowledge gap creates.

## 3. Methodology

**Figure 2:**
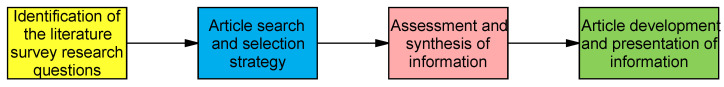
Methodology used in developing the literature survey presented in the present article.

Figure 2 provides a summary of the approach used to conduct and report the traditional literature survey presented in this article. Firstly, it was observed that the research field of eHealth system development is of importance to researchers and developers involved in the design and development of such systems, particularly in the wake of the COVID-19 pandemic and beyond. Therefore, the questions for the literature survey were prepared using certain selected keywords. This was followed by an appropriate article search and selection approach, evaluation and synthesis of the materials discovered, and reporting of our survey article. The details of the different key phases are presented in the subsequent subsections.

### 3.1. Literature Survey Questions

In order to design an eHealth system, it was vital to first define what an eHealth system is and what it consists of. In this regard, a search of the scholarly literature was conducted to discover existing survey studies on eHealth systems and their constituent components. The results of our overall review of eHealth and survey articles on the different constituent components of eHealth systems are detailed in Section 2. However, while many survey articles exist with regards to a number of constituent building blocks of an eHealth system, nevertheless, it was discovered that the survey literature had only minimal synthesized information on three critical components, namely: suitable architectures for the deployment of eHealth systems, the most relevant advances in mHealth, and security solutions for eHealth systems. This sufficed as a gap in the literature survey, which we sought to explore.

In this regard, the following research questions were formulated for our literature survey:

1. What are the most feasible architectures for establishing eHealth systems, together with their advantages and disadvantages?
2. How are these architectures implemented for eHealth systems?
3. What are the fundamental components and current improvements in mHealth that are necessary for the effective implementation of eHealth systems?
4. What are the relative advantages and disadvantages of these various mHealth elements in relation to eHealth systems, and how has the literature evolved in this regard?
5. What are the typical threats, goals, and solutions associated with the successful implementation of eHealth systems?
6. What are the research challenges and possible future prospects for the development/enhancement of architectures, mHealth, and security concerns in eHealth systems?

Following the development of these keyword-based literature survey questions, a second pre-literature search was conducted to determine whether these questions had been appropriately addressed and synthesized in other related survey publications. However, these questions were deemed worthy of synthesis, having confirmed that they were sparsely (often not) covered in existing survey articles (as documented in Section 2). Thus, this led to the next stage, which was the article search and selection phase.

### 3.2. Search and Selection Strategy

Our article selection criteria centered on research works dealing with architectures, mHealth, and security in eHealth systems. The following is an explanation of the search approach used to determine the selected papers:

1. We searched for articles using the Scopus, ACM Digital Library, IEEExplore, Springer Link, and Google Scholar databases. We considered the Scopus database because of its high-quality indexing and computer science-related information. IEEExplore, which focuses mostly on computer science, engineering, and electronics, received similar consideration. Due of their magnitude and potential to locate relevant papers, we also analyzed the ACM database and Spring Link. Following our exploration of these databases, we performed a last double-check using the Google Scholar database to decrease the number of missing articles.
2. Then, the terms that define our area of interest, namely “architectures”, “mHealth”, and “security”, were identified. These keywords were derived from a preliminary literature search to locate survey publications that had previously addressed the same issues. In addition, we generated a list of search strings that combine the operators “AND” and “OR” with the keywords and the term “eHealth”.
3. These keywords and phrases were used to search databases as mentioned above such as Scopus and Google Scholar, among others that were considered.
4. The search yielded over 22,700 results, which were then reduced based on the time span covered within the previous two decades. Additionally, these results were enhanced based on the following key categories: “architectures”, “mHealth”, and “security”. These keywords were used to manually reduce the number of articles to 250. The excluded articles were those that did not directly contribute to our area of interest.
5. In addition, survey papers located within this limited list were filtered and assessed to determine the uniqueness of our present article; and our findings are discussed in the related literature survey section (Section 2).

After obtaining the initial documents following the above search process, we evaluated their quality and began the assessment and synthesis of the acquired articles.

### 3.3. Assessment and Synthesis of Information

To analyze the quality of the retrieved documents, we set a few inclusion and exclusion criteria to improve our research methodology. These specifications are as follows:

- Inclusion criteria
  1. All articles must be published in journals or conference proceedings.
  2. All relevant survey articles must be very specific and pertinent to the existing elements of an eHealth system, and
  3. Articles relating to the given keywords must emphasize them extensively, as opposed to merely mentioning them.
- Exclusion criteria
  1. All articles without a full text were excluded,
  2. Articles that only mentioned the keywords were disregarded, and
  3. Preprints, reports, lecture notes, and proposals were removed.

After applying these inclusion and exclusion criteria to prune the identified articles, we further evaluated their quality as follows:

1. We generated a set of questions and answers to evaluate the contextual information of each article.
2. First, does the article primarily discuss eHealth system architectures? If yes, then the article was accepted to be studied. If no, was the discussion of architectures across a complete section? If yes, the article was studied; if no, it was deemed a simple mention of the term and was thus not considered worthy to be referenced.
3. Likewise, for each keyword (i.e., mHealth and security in eHealth), the same assessment questions as above were adopted to ensure that the retrieved articles were relevant enough for further synthesis.

Then, our goal being to construct a traditional literature review article, we next proceeded to examine each of the 250 articles, we synthesized the information within each article in relation to the research questions, and then discussed an overview of the gathered contextual information. Essentially, following further studying of the retrieved articles, we determined that 212 of the 250 articles were worthy of referencing in our article.

### 3.4. Article Development and Presentation of Information

After gathering and synthesizing pertinent contextual information around each keyword and research question, we then developed and improved the structure of our article. In this regard, the following strategy was adopted:

1. Following a modification of the well known IMRAD framework (i.e., Introduction, Methods, Results, and Discussion), the outline for our manuscript was constructed. In our case, since we aim to present a traditional literature survey article, the body of the manuscript was separated into three main sections based on the three keywords. Utilizing this strategy assisted in elucidating the scope of our article. However, in the absence of a results and a discussion section, we introduced the summary per section as well as the closing research challenges and future direction section as part of the body of work.
2. The different pieces of our synthesized information were then clustered depending on how each article relates to the research questions, and then each section was expanded upon to include the pros and cons of the different eHealth-related methods.
3. The final draft of our manuscript was then revised in accordance with the overarching purpose, which was to give a comprehensive overview of architectures, mHealth, and security in order to facilitate the development of viable eHealth systems.

## 4. eHealth: An Overview

This section offers a basic overview of the idea of eHealth in general, as well as the essential components (or building blocks) of an eHealth system. To achieve this, the section is organized as follows: first, we provide notable definitions and views of eHealth, followed by the various services anticipated from an eHealth system. Having established these contexts, we then briefly discuss each block, including the benefits of such blocks, in order to offer an intuitive grasp of the various aspects of eHealth.

### 4.1. Definition of eHealth

According to [27], eHealth is a new branch of medical informatics that refers to the use of the Internet and related technologies to organize and provide health services and information. eHealth encompasses, in a broader sense, the application of information and communication technologies to healthcare. It consists of all digital health-related data, including products, systems, and services. The term “health” in eHealth encompasses public health in addition to medicine, disease, and healthcare provisioning. The adoption of eHealth services aims to achieve a variety of objectives, including increased efficiency in healthcare, enhanced quality care, evidence-based medicine, empowerment of consumers and patients by broadening the knowledge base of medicine, encouragement of new relationships between patients and health professionals, education of physicians and consumers, enabling information exchange and communication, expanding the scope of healthcare, and a reduction in the cost of healthcare [15]. In brief, it promotes the sharing of health information, ensures effective healthcare, and enables health consumers to manage their own health. The goal of eHealth is to transform the healthcare system from a “provider-centric” model to a “patient-centric” model [2].

### 4.2. Major Components of eHealth

**Figure 3:**
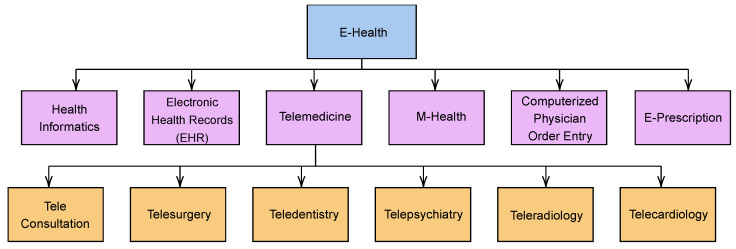
Major components of eHealth.

Recent advances in computerization, data digitization, and digital networks have facilitated the rapid development of eHealth systems and services [28]. Currently, eHealth includes a wide range of services and systems at the intersection of healthcare and information technology. These include telemedicine, a remote healthcare delivery system that utilizes telecommunications and information technology; electronic health records (EHRs), which contain electronic health information about a patient or person; and consumer health informatics, the use of medical informatics to analyze consumer needs [29]. Other areas include health knowledge management, which aims to capture, describe, organize, share, and apply healthcare knowledge; medical decision support systems, which are interactive expert systems that assist health professionals with decision-making tasks; and mHealth, which utilizes mobile devices for a variety of healthcare applications. Furthermore, eHealth comprises many other areas, as shown in Figure 3, and we provide a brief summary of the key characteristics of each component below.

#### 4.2.1. Health Informatics

Health informatics refers to the innovative use of the concepts and technologies of the information age to improve health care and well being. According to [30], health informatics encompasses the collection, analysis, and transmission of health data and information to support health care. The World Health Organization (WHO) defines it as “an umbrella term used to encompass the rapidly evolving discipline of using computing, networking, and communications—methodology and technology—to support the health-related fields, such as medicine, nursing, pharmacy, and dentistry” [31]. Notably, health informatics was only made possible by the interconnection of computers to form networks for information transfer. Thus, these networks served as a framework for connecting hospitals, and in the era of artificial intelligence, a vast array of services has become available.

In summary, despite the growth of health informatics as a field of study, the objective remains the same: To use the information gathered and its insights to achieve the following:

1. Enhance both individual and clinical patient care.
2. Help improve the health of global populations (such as using data for prediction and prevention of disease outbreaks).
3. Make it possible for organizations that provide medical care to do so at a lower cost.

There are many articles on health informatics, and we refer interested readers to the following references for more information: [32,33,34,35].

#### 4.2.2. Electronic Health Record

An electronic health record (EHR) is a comprehensive digital record of a patient’s health care history. It contains all of a patient’s health information that can be accessed electronically by healthcare providers. As a result, an EHR improves the precision, validity, and quality of the information contained in a health record. EHR improves access to information, allowing all healthcare professionals to share it readily in real time [28]. Through the use of EHRs, the constant availability of health information for patient care enhances the quality of care rendered to patients. EHRs have resulted in a paperless environment and eliminated many of the problems associated with paper health records. The goal of EHRs is to protect patient privacy and confidentiality while reducing medical errors and costs [26].

An EHR contains basic patient information, a record of all patient visits, diagnostic findings such as radiology images, diagnoses, and procedures performed, a lifelong medication record, and personal risk data such as allergies, vaccinations, and clinical referral letters. Medical records must contain information on all inpatients, outpatients, accident and emergency patients. A centralized system should be maintained by medical record systems, wherein all patient medical records must be kept, including admissions information, accident and emergency records, outpatient notes, and discharge lists. If the patient’s medical record cannot be located or has been lost by the EHR system, duplicate records can be created and merged with the older records.

An individual’s health record should typically be made securely accessible online by authenticated healthcare practitioners from a variety of distinct, interoperable automated systems within an electronic network. Consequently, for an EHR to support this functionality, the following five components would be necessary:

1. Person identifier
2. Faculty identifier
3. Provider identifier
4. Health information
5. Administrative information

Person identifier is a universal code that uniquely identifies each person in the health care system. A faculty identifier represents any institution or center that provides services inside the health system. Each health care provider inside the health system is identified by a universal provider identifier code. Diagnosis, X-rays, and prescriptions are examples of health information in a standard format that result from interactions between patients and health care providers. Administrative information, such as billing information, must be standardized for management objectives. The successful and efficient deployment of EHRs is enabled by the combination of these identifiers and the provision of protected database services. The articles in [35,36,37] provide further information regarding the specific technologies and platforms necessary for the realization of EHR systems.

#### 4.2.3. Telemedicine

The origins of modern telemedicine date back roughly a century to the invention of the traditional telephone [38]. Physicians provided medical advice over the telephone. The term “telemedicine” simply refers to the provision of medical services using telecommunications [39]. The prefix “tele” is a Greek word, which means “distance”. Therefore, telemedicine is the provision of medical services over a distance. Medical applications of telecommunications can be categorized as the transmission of medical data between transmitters and receivers. So-called “medical information” can be as simple as a doctor providing a consultation or as complex as body-specific data [40].

**Figure 4:**
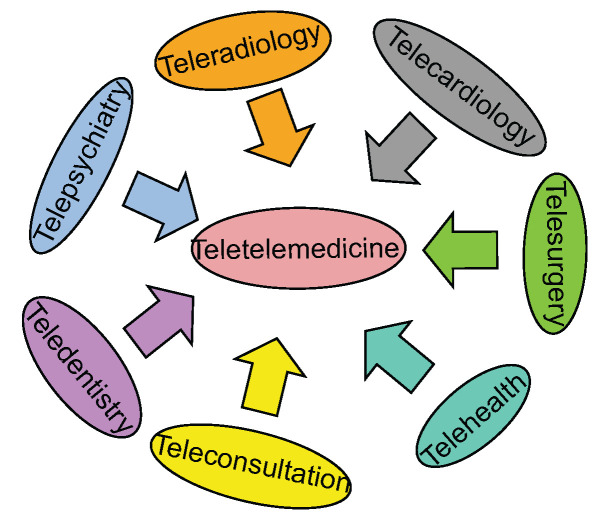
Branches of telemedicine.

There are three primary types of telemedicine: store and forward, remote monitoring, and real time interactive services [41]. Store and forward telemedicine is a method of telemedicine in which the information is initially stored by the sender and then forwarded to the receiver at their convenience. The option of remote monitoring telemedicine refers to the practice of using various technical devices to check on the health of a patient and provide clinical indicators about them remotely. In real time telemedicine, the sender and recipient are both online at the same time and pass live information back and forth between each other. On the other hand, there are other branches of telemedicine, which are categorized as telecardiology, teleradiology, telepsychiatry, teledermatology, telepathology, telesurgery, teleophthalmology, teledentistry, and general telemedicine, among others. Figure 4 illustrates the various branches of telemedicine.

#### 4.2.4. Computerized Physician Order Entry

Computerized physician order entry (CPOE), also known as computerized provider order management (CPOM), is the electronic entry of a physician’s orders for the treatment of patients (particularly hospitalized patients) under their care [42]. Additionally, it may be used to electronically request diagnostic testing and treatment purposes. The submitted orders are transmitted through a computer network to the medical staff or the departments (pharmacy, laboratory, or radiology) tasked with fulfilling the request. CPOE decreases the time required to distribute and complete orders while enhancing efficiency by reducing transcribing errors and preventing duplicate order entries, as well as by expediting inventory management and billing services [43].

Although CPOE and computer-based patient record (CPR) are frequently used interchangeably, they are actually quite distinct. CPR is defined as a collection of patient-specific health information linked by a patient identifier [44]. CPR could involve as little as a single episode of care for a patient or as much as an extended period of healthcare information. Early CPR was primarily concerned with functions such as medical alerts, medication orders, giving integrated data on a patient’s registration, admission, and financial details, and recording information from nurses, laboratory, radiology, and pharmacy. Although this type of CPR was used in a number of contexts, the focus was limited to inpatient hospitals for the exchange of health information.

In a general CPOE system, the representation of an order sequence would contain certain information that should be displayed in clear text to a CPOE system personnel with the following content [45,46,47]:

- Specific details pertaining to the patient in question
- The function of a required member of the staff.
- The resources, materials, and medication given.
- The procedures that are to be carried out.
- The proper order of operations that must be followed.
- The feedback to be taken into account.
- The documentation unique to each individual case that must be constructed.

In general, CPOE is beneficial since it is capable of better organizing historical information and it is designed in a manner that is comparable to those of conventional hospital information systems. The primary advantage of CPOE is its capacity to transfer information from the physician responsible for the treatment of a specific patient to the various personnel responsible for processing the treatise itself [48]. This makes CPOE the primary instrument for information transfer to the personnel who are actually conducting the work, and it also helps reduce the workload for the personnel who are liable for accounting. Consequently, the demand for precise accounting is promptly addressed through the provision of feedback on the conclusion of orders. CPOE offers a number of benefits, the most important of which are as follows [49]:

1. Reduce errors and enhance patient safety: At the very least, CPOE can assist an organization in reducing errors. This is accomplished by ensuring that providers produce orders that are standardized, clear, and comprehensive. In addition, CPOE technologies typically incorporate clinical decision support tools that are already built in. These tools enable the technology to perform an automatic check for drug interactions, pharmaceutical allergies, and other potential issues.
2. Improve efficiency: CPOE can help an organization improve its efficiency by accelerating the delivery of medication, laboratory, and radiology orders to pharmacies, facilities that perform radiology, and laboratories, thereby reducing the amount of time wasted and increasing the amount of time available for other tasks.
3. Improve reimbursements: Some items require pre-approval from insurance schemes. When CPOE is integrated with an electronic practice management system, it has the ability to highlight orders that need pre-approval, which can help you reduce the number of insurance claims that are rejected.

#### 4.2.5. E-Prescription

Electronic prescription, also known as e-prescribing or e-prescription, is a technology framework that enables physicians and other medical professionals to write and submit prescriptions to a participating pharmacy electronically rather than using handwritten or faxed notes or calling in prescriptions [50].

An e-prescribing system can be thought of, at its most fundamental level, as an electronic reference handbook. In this case, the software and systems for electronic prescribing can even function as a standalone prescription writer. They are able to generate and refill prescriptions for individual patients, manage medications and examine patient histories, establish a connection with a pharmacy or other sites that dispense drugs, and integrate with an electronic medical record (EMR) system [51].

Nowadays, medical professionals, including doctors, nurses, and other health practitioners, are increasingly reliant on the use of computers to process patient records, prescriptions, and appointment scheduling. As a result of this high rate of computerized device adoption, electronic prescriptions, EHRs, and e-pharmacies are just a few of the mainstream digital solutions that will continue to be widely used in the healthcare industry [52]. Furthermore, it is envisaged that in the recent wake of artificial intelligence systems, the potentials for electronic prescribing will greatly expand, towards ultimately minimizing the number of errors committed in medical prescribing [53].

## 5. eHealth: An Overview of Architectures

**Figure 5:**
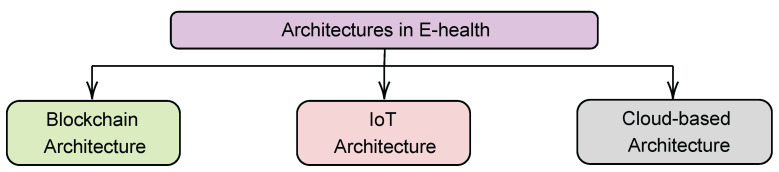
Different architectures for deploying eHealth.

In this section, we cover different architectures that are feasible options for deploying eHealth systems. For the success of any eHealth system, the necessity for an architecture is crucial. As a result, we review three contemporary architectures noted in Figure 5. This section is organized as follows: Firstly, we explore each architecture in distinct subsections, emphasizing the strengths and limits of each. In addition, we compare blockchain and cloud-based architectures in order to equip developers with the knowledge required to make informed design decisions. We also provide a synopsis of other architectures that have been referenced in other studies. Then, the section is concluded with a summary of what the literature covers well and what it does not cover well, as well as the key ideas of this section.

### 5.1. Blockchain-Based Architecture

In order to understand the term “blockchain”, it is imperative that we understand the concept of a distributed ledger. According to [54], a distributed ledger (also known as a shared ledger) “comprises of a consensus of replicated, shared, and synchronized digital data that is distributed together with a group of nodes, operating as a distributed database, generally geographically dispersed”. Therefore, a blockchain is a specific kind of distributed ledger that was conceived by Satoshi Nakamoto in 2008 and is used as a fundamental component of the digital currency Bitcoin [54,55]. The data stored in a blockchain needs to be incorruptible, which can be achieved through the application of cryptography, as well as through the use of digital signatures and digital fingerprints (i.e., hashing) [54]. Additionally, it is necessary to ensure that there is consensus among the peers (transacting parties), taking into account the possibility that some of the peers may be providing inaccurate data, and that some or all of the peers may be experiencing problems with their computers or networks, or even that some parties may be engaging in malicious activities by attempting to subvert the ledger [54].

**Figure 6:**
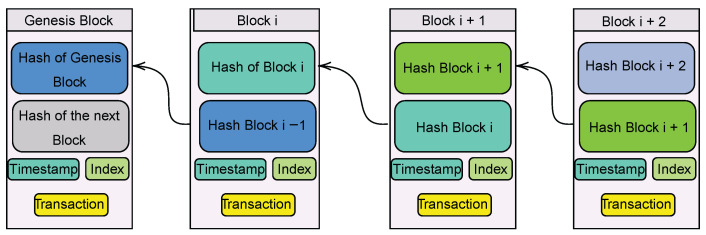
A representation of blocks in a typical blockchain process.

Therefore, as noted, a blockchain is a distributed digital ledger that records transactions in a sequential order using blocks. Each block in the chain has its own copy of the previous block’s data. Every one of these transactions has been given a unique digital signature by the entity that is responsible for making them. The blockchain is created when individual transactions are grouped together into a block and then added to the chain for permanent storage. The hash of the prior block is included in each new block, and this information is passed along the chain until it reaches the first block, which was produced when the blockchain was initially constructed and is known as the genesis block [54]. Then, we can consider that a blockchain functions as a state transaction system (state machine), in which there is a state that corresponds to the snapshot of the chain (the result of all transactions up to this point), and after adding a new block of transactions to the chain, we obtain a new snapshot that corresponds to a new state of the system, as a result of the new transactions [54] (see Figure 6).

There are three major types of blockchain: public, fully private, and consortium blockchains [54]. Public blockchains (e.g., Bitcoin) are a type of blockchain in which anybody can view and send transactions and expect these transactions to be included in the blockchain if they are genuine, and, furthermore, anyone in the world can participate in the consensus process [56]. Fully private blockchains are blockchains in which the write permissions are kept centralized to one organization (even if they are scattered across facilities), and these permissions exist within a closed set of players who are already known to one another (for example, a supply chain) [54]. Finally, consortium blockchains are somewhat private in the sense that the process of reaching consensus is managed by a number of different sets of nodes that have been chosen in advance [57]. The right to query the blockchain in this kind of blockchain can either be made available to the public or kept private to the members.

Blockchain technology has recently found application in the field of eHealth following advances in the development of biosensors. Many wearable sensors, such as those used to monitor blood glucose levels, heart rate, body temperature, and blood pressure, are now connected via IoT networks, thus allowing medical professionals and other healthcare institutions to access such data remotely and automatically. Furthermore, it is expected that patient information will typically be stored in a secure location on a server and handled off-site. In light of this development, patients can understandably be concerned about the privacy and confidentiality of their data. This is because multiple security breaches are possible in such circumstances. An adversary, for example, could intercept healthcare data while it is being transmitted over the Internet, alter it, and inject incorrect data into healthcare data centers. Furthermore, such attackers may steal data from remote servers.

Following the above, the authors of [58] provided a comparison of blockchain and IoT/cloud services, as represented in Table 2. It can be noted that blockchain systems use a decentralized technology and that data stored is incorruptible, secure, and does not need to pass through a central server. As a result, these characteristics serve to justify the need to develop blockchain-based eHealth architectures.

Regarding the important inferences from the literature, we underline first that the study and development of blockchain technology has expanded nearly rapidly, as evidenced by the vast amount of information available in the literature. In relation to eHealth systems, however, the authors of [59] noted that the primary benefit of blockchain technology in healthcare systems is the management of patients’ electronic medical records. Consequently, several initiatives have been carried out to enhance blockchain technology for medical applications. For instance, the authors of [60] presented a blockchain-based healthcare system that allows efficient authentication of EHR data validity and signer identification. The results of their experiments revealed that the proposed approach is effective and feasible. Similarly, ref. [61] presented a blockchain-based safe and privacy-preserving health information (PHI) exchange strategy for diagnostic enhancement in eHealth systems. Specifically, the authors created a private blockchain for the storage of PHI, and a consortium blockchain was proposed for the secure indexing of PHI information. They were able to demonstrate that the suggested protocol can satisfy the security objectives of eHealth systems by applying such a technique. The literature on the creation and upgrading of blockchain technology for eHealth systems is fairly extensive, and this topic has been addressed in a number of prominent survey publications, such as in [59,62,63]. Most importantly, these publications often note that blockchain technologies need to be improved prior to their adoption in eHealth systems. More specifically, this improvement should focus on optimizing the computational and memory resources that are available. This is due to the fact that eHealth systems are frequently mission-critical real time systems that cannot tolerate the huge latency limitations that blockchain technologies frequently create.

**Figure 7:**
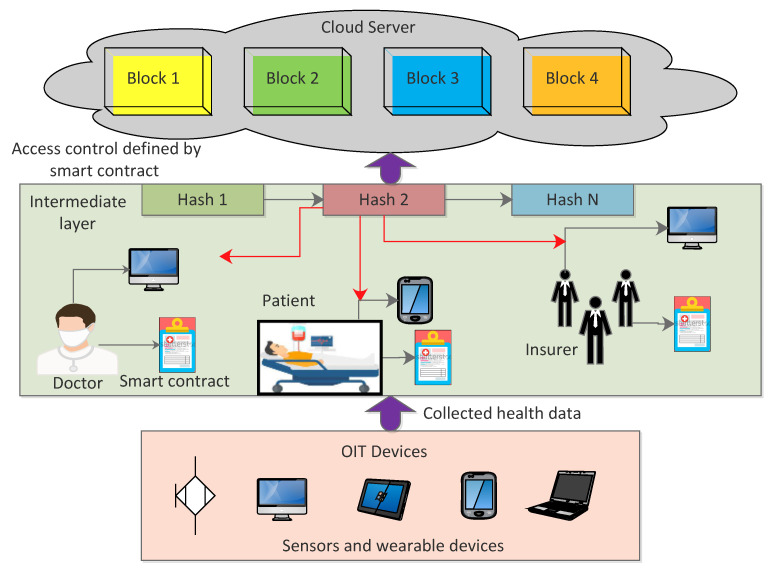
Blockchain Architecture.

Furthermore, in terms of the technical details of a typical blockchain architecture, Figure 7 suffices in this regard. The first layer of the architecture consists of IoT devices, such as sensors and wearable devices. The data collected from this layer are sent to the intermediate layer, which is comprised of doctors, patients, insurers, and researchers, with each device defining its own smart contract. After transactions are created, blocks are constructed, and consensus protocols are executed, the blockchain is stored on a cloud server and a hash of each block is returned. Every node at the intermediate layer simply stores a chain of block hashes. The cloud is centralized; thus, if there is any change to the block data, the hash of that block will change, as would the hashes of all other blocks stored in the intermediate layer. This aids the architecture in keeping track of any modifications made to the data.

#### 5.1.1. Benefits of the Blockchain-Based Architecture

A blockchain-based architecture poses the following benefits:

1. It ensures that EHRs are stored in a secure and open manner.
2. Provides regulated controlled access to data.
3. Ensures the integrity of the data and that they cannot be changed.
4. Makes it possible to share data.
5. Provides remote patient monitoring.
6. Adapts to the challenges posed by constrained IoT devices.

#### 5.1.2. Limitations of the Blockchain-Based Architecture

1. Several articles have discussed the drawbacks of blockchain technology in the context of eHealth. For example, in [64], the drawbacks of proof-of-work (POW) in a consensus blockchain algorithm were highlighted. To add a block to a POW-based blockchain, miners must perform computationally expensive tasks (carried out by multiple entities), making Sybil attacks nearly impossible. Miners must then be able to perform a certain amount of work in order to calculate the number. When a miner solves a problem, all other nodes must verify that the solution is correct. As a result, POW consumes more energy, rendering it inefficient for use in low-power applications. Furthermore, the increase in block transactions does not correspond to an increase in POW nodes participating in block verification; thus, it is not scalable.
2. Another disadvantage mentioned in [64] is that the blockchain mining process benefits the wealthiest participants, who may own a larger stake than other nodes.
3. In a different article [65], the authors stressed the importance of the determination of a data sharing protocol as a difficulty in the implementation of blockchain technology in eHealth. For instance, there is a need for clarification regarding how a patient can pick which data to disclose and with whom they share it. The patient, who is the legal owner of the information, is the one who needs to give permission for a healthcare provider to access it. It is not apparent who has the authority to act on behalf of a patient in the event that the patient is unable to carry out the requested action for any reason. There is also a lack of clarity on the quantity of health data that must be stored online and whether or not that data may be shared indefinitely or for a predetermined period of time.
4. In addition, a notable limitation of the blockchain-based architecture is the difficulty of achieving both cost-effectiveness and scalability while managing vast quantities of data that have not yet been subjected to quality assurance testing in production settings. When the volume of traffic increases, the length of time it takes to complete a transaction might become prohibitively long, depending on the protocol; this has an effect on the scalability of the system and the amount of computing power that is necessary.

### 5.2. IoT-Based Architecture

Prior to introducing a reference IoT-based architecture, it is essential to comprehend the general idea of IoT. IoT is defined as “the interconnection of heterogeneous devices that can be controlled and adjusted remotely over a wireless infrastructure in order to eliminate human interactions and increase productivity” [66]. In another article [67], IoT is defined as a collection of computing devices that can monitor and transmit data from an environment over the Internet in order to provide consumers with services and information. The inference from these definitions is that IoT is primarily concerned with the remote control, monitoring, and transmission of data from one device to another over the Internet. These devices are referred to as smart objects or “things” and they are able to share data and information from the monitored environment. Smart objects typically have limited processing power and a lower level of security than personal computers and smartphones.

The aforementioned characteristics of IoT devices have made it necessary to investigate methods that are both effective and low-computing in nature for the purpose of access control and data privacy in IoT applications [68]. As a result, IoT systems have found applications in a variety of fields, including the transportation sector, industry, education, the healthcare sector, and smart applications. In the realm of eHealth, the integration of IoT is geared towards enhancing the business procedures that are carried out by healthcare organizations, professionals, patients, and consumers in order to improve the overall health condition of patients. Thus, we will examine the function, integration, and impacts of IoT in eHealth.

#### 5.2.1. IoT in eHealth

The use of IoT applications in the medical field has enormous potential. For example, hospitals will be able to improve patient care and management by increasing their insight into the numerous flows and activities that take place within their facilities. IoT applications are also relevant from the perspective of traceability of the drug circuit outside of the hospital. Through the use of linked pills or connected medical packaging, the various players along the care pathway will be able to determine whether or not a patient is actually taking the medication that has been prescribed to them. It also has the potential to provide solutions to a number of issues that exist within the healthcare industry, including hospital management and the monitoring of patients in a real time manner. In recent years, a number of IoT applications have been noted, a few of which are mentioned as follows:

- Patient monitoring: for example, tensiometer sensors have been implanted in hypertensive patients as part of a real time monitoring framework in order to reduce time-consuming and inconvenient follow-up visits to the doctor [69].
- Preservation facilities: for example, medical refrigerators have been designed with IoT devices to control the conditions inside freezers for storing vaccines, drugs, and organic elements [70].
- Elderly home tracking systems: Doctors can monitor elderly patients at home, lowering hospital costs and increasing time intervention to crisis situations [71].

Following the discussion of the various application areas and benefits of IoT systems in eHealth, we will then shift our focus to the different IoT-based architectural frameworks.

#### 5.2.2. IoT Architectures for eHealth

**Figure 8:**
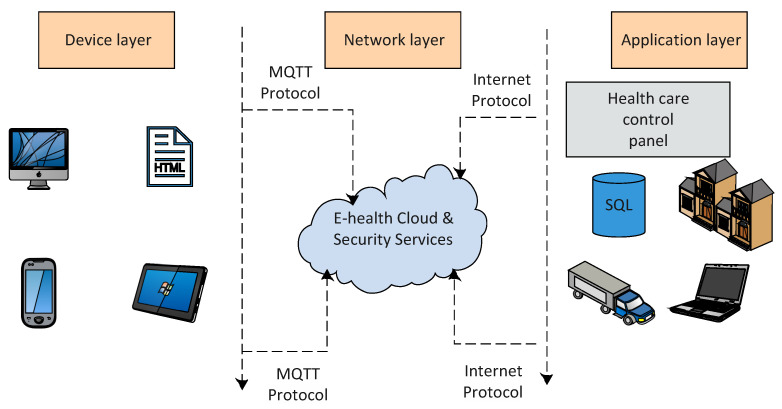
Three-layer IoT-based architecture.

Figure 8 depicts a typical reference IoT architecture, which consists of three primary layers. The first layer is known as the device layer. It is comprised of several intelligent devices fitted with sensors to gather and analyze data in accordance with the initiative for big data analysis. The network layer is the second layer. It contains all networking, routing, and identification technologies required for the application’s functionality. The third layer is the application layer, which is responsible for offering services to consumers, such as requesting the temperature of a certain place from a sensor. At this layer, data analysis can be performed on a bigger scale. In addition, the application layer enables the analysis of data in the cloud in order to provide advice for patients with urgent conditions (see Figure 8).

**Figure 9:**
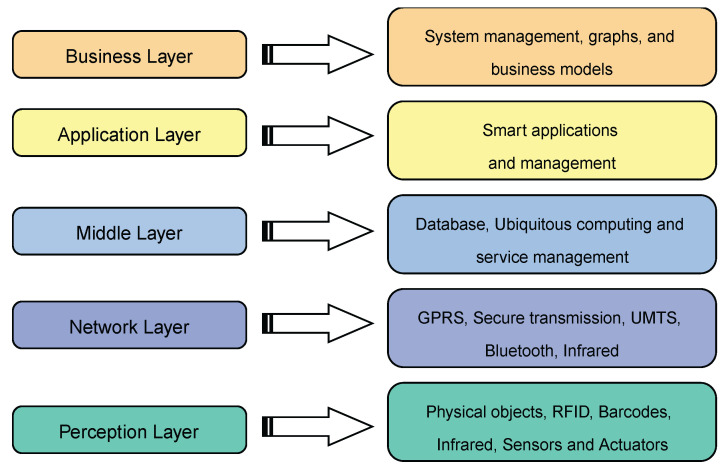
Five-layer model of an IoT-based architecture.

In addition to the three-layer model, there is also a five-layer IoT model, briefly discussed as follows:

1. Perception Layer: The perception layer is similar to the device layer in the three-layer model. It includes physical objects and sensor devices. Depending on the mechanism used to identify the object, the sensors may be based on RFID, 2D-barcode, or infrared technologies. This layer is primarily concerned with the identification and gathering of object-specific data by sensor devices. Depending on the type of sensors, the data may pertain to location, temperature, direction, motion, vibration, acceleration, humidity, chemical changes in the air, to name a few. The acquired information is subsequently transferred to the network layer for transmission to the information processing system in a secure manner.
2. Network Layer: This layer is often referred to as the transmission layer. The network layer delivers data from sensor devices to the information processing system in a secure manner. Depending on the sensor devices, the transmission method may be wired or wireless, and the technology may be 3G, UMTS, WiFi, Bluetooth, infrared, ZigBee, etc. Consequently, the network layer sends data from the perception layer to the upper layers.
3. Middleware Layer: IoT devices implement a distinct type of service where each device only connects and communicates with other devices that support the same service type. The layer responsible for facilitating these tasks is called the middle layer, which is in charge of service management and it is connected to the database. It stores the information received from the network layer in the database. It conducts information processing and ubiquitous computation and makes judgments automatically based on the outcomes.
4. Application Layer: This layer enables global application administration based on the middleware layer’s processing of object information. IoT applications at this layer include smart health, smart farming, smart homes, smart cities, and intelligent transportation, among others.
5. Business Layer: This layer is responsible for managing the IoT system as a whole, including applications and services. Based on the data received from the application layer, it constructs business models, graphs, flowcharts, etc. The true success of IoT technology is contingent upon sound business strategies. This layer will help identify future actions and company strategies based on outcomes analysis. These five layers are summarized in Figure 9.

In terms of important technological efforts in establishing IoT-based architectures for eHealth systems, the authors of [66] discussed current IoT-based designs in the literature and also proposed a three-layered architecture. These layers consisted of a perceptron, a network layer, and an application layer. The authors’ qualitative comparison of their architecture to existing ones led them to the conclusion that their design allows for the integration of fog, blockchain, and light-fidelity (LiFi) technologies, whereas others do not. In a similar manner, the authors of [72] adopted a three-layer design consisting of the device, network, and application layers, as previously described in this section. However, they further focused on illustrating how wearable sensors may be included in the design of their architecture and concluded that their suggested framework is capable of achieving this objective. In contrast, the authors of [73] investigated IoT architectures by integrating Big Data analytics. They concluded once more that the fundamental architectural framework of an IoT system should consist of a device, fog, and cloud layer. In this instance, the fog layer corresponds to the network layer of Figure 8, where processing and network linkages occur. Their conclusion was that due to the rapid rise of the Internet of Things and the popularity of wearable devices, caution must be taken when implementing these technologies due to valid concerns of consistency, safety, and cost-effectiveness. In addition, they underline that incorporating Big Data analytics into eHealth infrastructures can significantly enhance eHealth services for healthy lives.

#### 5.2.3. Benefits of IoT-Based Architectures

Listed below are few benefits of IoT-based architectures:

1. Patient safety and quality of life can both be improved by continuously monitoring patients’ conditions without interfering with their daily lives and allowing for remote assessments.
2. Individuals will have a greater level of autonomy and initiative with regard to their own health status, which will provide them with a better level of control over their own well being.
3. By analyzing a massive amount of data, health experts can improve preventative care and make the eHealth system more effective.
4. It is possible to lower the expenses of patient care provided in hospitals and prevent supply shortages by employing remote monitoring and automated equipment stock management.

#### 5.2.4. Limitations of IoT-Based Architectures

1. Keeping the sensitive data collected and transmitted by IoT devices secure is difficult as their use expands and evolves. Despite the importance of cybersecurity, IoT devices are not usually incorporated in the plan. Devices must be safeguarded against physical manipulation, Internet-based software assaults, network-based attacks, and hardware attacks.
2. Although it may appear that IoT devices perform simple functions, such as tracking a patient’s temperature, there is a great deal of technical technology involved in their creation. In addition, if they provide erroneous vital data to another workflow or system, they may negatively impact everything associated with it. Inaccurate measurements can be devastating and may be difficult to detect and correct.
3. In order for a lot of different IoT devices to work correctly, they need to be connected to the Internet and have constant electricity. If either fails, the gadget as well as anything else that is attached to it will become inoperable. When it comes to today’s enterprises, IoT devices are so interconnected that if they go down, everything can come to a grinding halt. As a result, there is a need for gadgets that are powered by batteries, which raises further concerns about energy management and sustainability.
4. Because there is presently no consensus over IoT eHealth-based protocols and standards, it is possible that devices manufactured by various companies will not be compatible with the technology that is currently available. It is possible that each one will require a distinct configuration and connection to the hardware, making it difficult to deploy efficiently.
5. The deployment of sensitive eHealth IoT devices and systems typically calls for significant investments of both time and money. There are a lot of devices that need to be bought and set up, as well as employees who need to install them, others who need to integrate them into the network, and support calls that need to be made to the manufacturer. Health businesses are able to quickly recoup their losses when all of their operations are consolidated into a single location. However, the cost can be expected to increase if the health company or institution decides to distribute them.

### 5.3. Cloud-Based Architecture

A typical cloud-based architecture allows a healthcare institution, such as a hospital or clinic, to manage data acquired via wireless sensor networks (WSNs) for patient monitoring [74]. Such a system is expected to be scalable and capable of storing massive amounts of data collected by sensors. Furthermore, according to [75], a cloud-based web architecture typically relies on a “home system” for the collection of information from a heterogeneous set of devices, provides a high-level description of the proposed overall architectural model, induces market opportunities, and provides a platform for use by healthcare application developers and service providers. Such an architecture should also include information on how the web server Application Programming Interfaces (APIs) are implemented for gathering, processing, and storing of data from WSNs.

The following are some of the components that make up the cloud-based architecture proposed in [74]:

1. The WSN, which is responsible for collecting patient information regarding their health.
2. The monitoring applications, which provide specialists in the healthcare industry with access to the data that have been stored.
3. The healthcare authority (HA), which is responsible for defining and enforcing the safety policies of the healthcare institution.
4. The cloud servers, which are responsible for ensuring the storage of data.

Similarly, in addition to points 1 and 2 above, the cloud-based architecture in [75] is made up of the following components:

1. Cloud computing and web services: The availability of a cloud-based service platform would make it much simpler to construct healthcare apps and services that make use of the vast health-related data set provided by end-user devices.
2. Home system: This system typically consists of a central controller that collects data from sensors and medical equipment used to monitor a person’s health and serves as a home gateway.
3. Web portal: The web portal acts as the system’s user interface.

**Figure 10:**
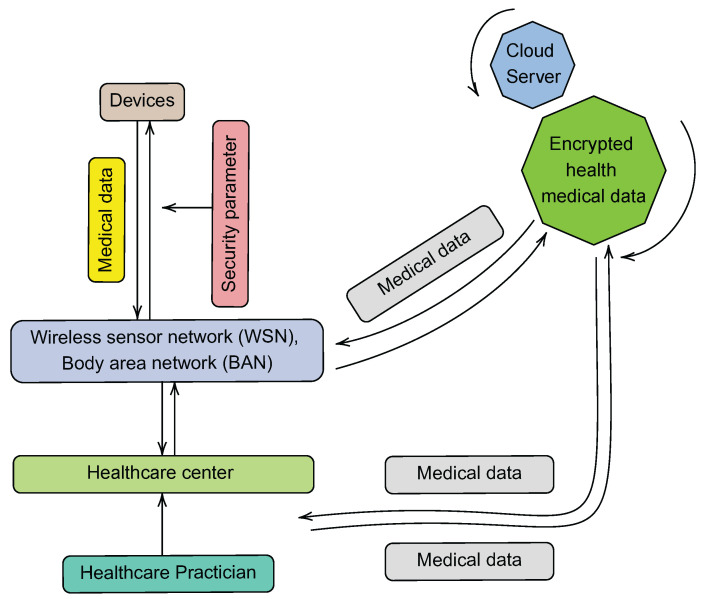
Cloud-based architecture.

Figure 10 illustrates the aforementioned characteristics of a typical cloud-based architecture. This design is comparable to the IoT-based architecture; however, it enables information to be uploaded to the cloud not only from the WSN level but also from the healthcare center level. There are many examples where typical cloud-based architectures have been deployed for eHealth purposes. For example, in [76], patients’ EHRs were standardized and linked together to create a scalable cloud-based EHR infrastructure. Following this architecture, stakeholders can obtain a patient’s EHR by submitting a single request to an integrated data cloud-based repository. In compliance with HL7-FHIR standards, the authors implemented a two-level OpenEHR method. They then used a preset set of axes and a scoring system to compare their architecture with five other architectures in the prior-art. With the aid of standards and cloud computing, they were able to demonstrate that their architecture was capable of integrating a wide range of EHR systems.

In [77], the authors developed an architecture that integrates cloud, edge, IoT, and other upcoming technologies. Their architecture includes a device layer in which wearable sensors collect patients’ health information. The subsequent layer is the multi-access edge computing layer, which provides computer resources for user applications and brings these resources closer to the users. The beyond 5G mobile network layer facilitates the straightforward integration of MEC with other 5G applications. Then, the multi-cloud infrastructure layer offers eHealth applications with large-scale and on-demand resources. These applications take a significant amount of computational resources, and as a result, the design tries to manage the many stages and lifecycles of such programs in order to ensure consistent system operations.

Another cloud-based architecture is described in [78], which provides a safe and enhanced cloud infrastructure for the transmission of eHealth data. Their cloud-based architecture comprises four layers: the master cloud server, slave servers, virtual subservers, and cloud users. Experimental results reveal that their proposed layered cloud architecture guarantees the trustworthiness of its implementation and establishment, as it makes the current architecture more lightweight, efficient, and safe for the transfer of e-health data.

#### 5.3.1. Benefits of Cloud-Based Architectures

1. Cost reduction: Cloud services rendered in a cloud-based architecture are capable of lowering the expenses associated with building up health-based infrastructure, maintenance, and utility management for the servers. Designers only pay for server time and memory space when using cloud computing services.
2. Reliability: Cloud-based architectures offer data redundancy because the data is not merely saved on a single server but rather is distributed across numerous servers.
3. Accessibility: eHealth is a highly accessible and flexible technology as a result of its ability to store information in a cloud-based architecture, which enables users to access the information regardless of the system they are using, whenever they need it, and wherever they are.
4. Large capacity for data storage: The cloud service provides users with an almost infinite capacity for data storage, which may be expanded at any moment for a very modest additional cost on a monthly basis.

#### 5.3.2. Limitations of Cloud-Based Architectures

1. Downtime: Cloud-based architecture, like all other architectures, is dependent on the availability of electricity and the Internet. When Internet access is lost, an architecture with this dependency becomes unreachable.
2. Security: Because data can be accessible by other people while they are in transit or while they are stored in the cloud, there is always a risk of the data’s confidentiality being compromised. The use of a cloud computing system indicates that the architecture has completely put faith in the security and confidentiality of the data being stored on cloud computing servers provided by third-party businesses. Consequently, users cannot necessarily sue the cloud service providers for inaccuracies in the data whenever there is a problem since such challenges are typically not covered in the terms of conditions.
3. Latency: Due to the obvious great distance that must be traversed to send data to the cloud, there is a problem of delay that emerges, which has the potential to disrupt emergency services provided by the eHealth system. Additionally, the quality of the servers used for cloud computing may also have an effect on the processing speed during peak times, which may result in latency in an electronic health record system.

### 5.4. Summary of Discussion

Architectures that are effective, efficient, and scalable are essential for the successful deployment of eHealth services. As a result, the development of such a reference architecture continues to be a major topic in the eHealth literature. This section has provided an overview of the three major types of architectures, which are blockchain-, IoT-, and cloud-based.

#### 5.4.1. Our Take-Aways

1. From our review, it is worth noting that one prominent and contemporary architecture is the IoT/cloud architecture, which is characterized by a three-layer model, namely the perception (device), network, and application layer. The perception layer is made up of several intelligent objects that are outfitted with sensors to collect and process data as part of the Big Data initiative [79]. The network layer being the second layer includes network, routing, and identification technologies required for application operations, while the application layer, which is the third layer provides services to users. The IoT/cloud architecture is singled out for mention here because it represents a fundamental framework upon which eHealth systems may be designed and implemented. This is because it meets the needs for eHealth services in terms of sensing, processing, and decision-interfacing.
2. Despite the fact that these architectures have been discussed separately, any realistic and viable architecture must integrate these three different technologies in order to ensure workability. Thus, a unified eHealth architecture is advocated that will incorporate blockchain, IoT, and cloud-based technologies.
3. A wide range of other architectures and technologies have been used to achieve this integration via the use of 5G, Fibre, and web services.
4. As shown in Table 3, the literature on architectures for the implementation of eHealth systems has expanded to include a wide variety of architectures with their specific purposes.

#### 5.4.2. Our Recommendations

1. In eHealth systems, a substantial amount of work has been covered in terms of developing specific designs for various objectives, such as continuous monitoring, remote administration, control, and decision making. However, meaningful comparative assessments of these various architectures in terms of their viability, compactness, and security have much to be desired and more research is needed in this regard.
2. We also recommend that future survey articles should cover additional topics involving the implementation of Big Data analytics into eHealth designs, which has witnessed tremendous research and could suffice as a standalone survey.
3. Furthermore, to summarize, we note that there may not be a single best architecture because they all have their respective benefits and drawbacks; rather, we suggest that a unified integrated architecture may be the way forward for the deployment of eHealth infrastructure and services.

## 6. eHealth: Advances in mHealth

In this section, we explore mHealth in relation to the realization of eHealth systems. Recent improvements in wireless sensors, mobile apps, and communication technologies have resulted in a fast expansion of the mHealth literature. Therefore, in order to organize our presentation, we adopt the following structure: First, as a foundation for comprehending its idea and expectations, we present briefly the various definitions of mHealth. Then, we discuss wireless sensors in mHealth, mobile computing devices, and the various mHealth communication technologies. These components of mHealth are deemed essential for developing mHealth solutions that are compatible with eHealth systems, hence their inclusion in this section.

### 6.1. Definition of mHealth

Mobile health (mHealth) is an enabling technology in the deployment of eHealth services that combines the evolution of emerging wireless communications and network technologies with the concept of “connected health care” anytime and anywhere [87,88]. In [89], mHealth is defined as the use of mobile telecommunications and multimedia technologies, as well as their incorporation into new mobile health care delivery systems.

**Figure 11:**
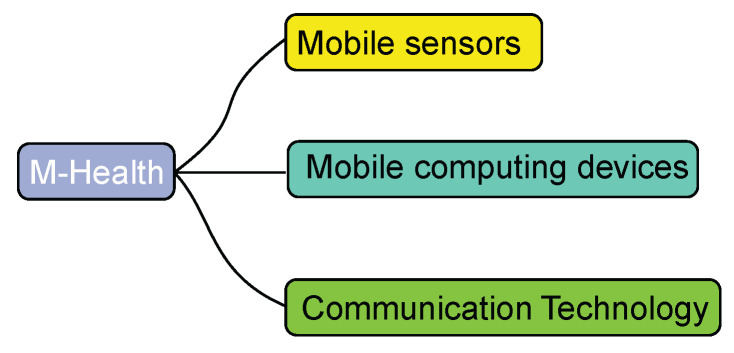
The different aspects of mHealth.

As noted, the term “mHealth”, which refers to the use of mobile wireless technologies for public health, is an essential component of “eHealth”, which is defined as the efficient and risk-free application of information and communication technologies in the service of health and fields related to health [90]. Nowadays, the term “digital health” is frequently used as an all-encompassing word that includes not only eHealth but also new subjects such as the use of sophisticated computing sciences (for example, in the domains of “big data”, genomics, and artificial intelligence) [88,89,90]. Therefore, mHealth can be subdivided into the following main areas: medical sensors, mobile computing/devices, and communication technologies deployed in eHealth as depicted in Figure 11. Each component is discussed as follows:

### 6.2. Wireless Sensors in mHealth

Wireless sensors in the medical domain are used to collect data regarding the current condition of patients as well as their medical history [91]. When it comes to certain chronic conditions, taking regular vital sign readings is not only an absolute necessity but also an essential component of achieving optimal treatment results. Thus, attention is often directed toward patients who require ongoing monitoring of their current state of health. According to [92], patient monitoring devices can be categorized into two distinct groups: alert monitoring and data acquisition devices. In the first category, the data from the patient are compared to the ranges or limitations that have been established beforehand. The system will then decide whether or not a patient needs assistance at this point. The second type of devices are intended to monitor a patient while simultaneously gathering and storing all data for the purpose of additional research and examination.

The miniaturization and autonomy of medical sensors has greatly enhanced the capability for mHealth towards providing medical monitoring, screening and even therapeutic actions. There is a wide variety of wearable sensors available, such as accelerometers and gyroscopes, smart textiles and actuators, wireless communication networks and power sources, and data capturing technology for the purpose of processing and decision support [93]. Having a device that a patient can wear lessens the limits that are placed on their mobility and the activities that they do on a daily basis, which enables monitoring in the environment of the patients directly at home as well as at work.

In terms of research endeavors in the literature, the findings presented in [94] illustrate the feasibility of integrating wearable devices and sensors in wireless sensor networks for the sake of eHealth applications. The authors developed a data gathering and monitoring application with applicability to the health of athletes. They concluded that the system can be easily adapted to a variety of eHealth applications with minimal modification. In [95], advancements in the application of wireless biosensors for eHealth monitoring in a novel cloud-based and service-oriented architecture were discussed. The authors presented a framework for the collection of real time patient data, noninvasive monitoring, and the deployment of management methods. It was demonstrated that the framework can reduce network overhead caused by sensors, mHealth applications, and backend system connections. Other recent research efforts have focused on the development of wireless biosensors for particular medical applications, such as temperature monitoring [96], standardization [97], and COVID-19 [98].

Furthermore, a summary of a few well known sensors for mHealth purposes is provided in Table 4. The most used and well known sensors are accelerometers and electrochemical sensors that measure acceleration of objects in motion along reference axes and provide basic step and activity counts used as a quantitative assessment of physical activity. For further information on sensors, self-tracking and monitoring, and their direct clinical applications, we refer interested readers to [99]. Other interesting areas of wearable biosensors include the use of machine learning algorithms in wearable sensors [100], biological age estimation [101], smartphone sensing [102], and IoT security for wearable sensors [103], to name a few.

### 6.3. Mobile Computing Devices in mHealth

Mobile computing is a technology that enables users to transmit data, audio, and video via devices with wireless connection from wherever they are [117]. This implies that mobile computing is best suited for mHealth applications due to certain governing principles of mobile computing, the most notable of which are portability, network connectivity of computer equipment, interactivity, and individuality [118]. By portability, mobile computing devices should be developed to easily move about with them. For network connectivity, there should be no downtime or lag in a mobile computing system’s network, regardless of the movement of the nodes that are connected to the network. Interactivity ensures that mobile computing nodes are connected to one another so that such nodes can interact with one another and work together via data transfers. By individuality, a mobile computing system must be able to adapt the technology in order to meet the specific requirements of each user and also to collect the relevant context information for each node.

Mobile computing devices and multimedia technologies in mHealth include:

- Mobile phones;
- Personal digital assistants (PDAs);
- Smartphones;
- Portable media players;
- Handheld and ultra-portable computers such as tablets PCs.

These devices make it possible to communicate via text messages, photos, and videos; they also provide access to the telephone and the Internet, and they can play back multiple types of media. Furthermore, WBAN personal server applications can run on wireless handheld devices such as smart phones (which are voice-centric devices with PDA-like data capabilities) or WWAN-enabled PDAs or personal communicators (which are data-centric devices with voice capabilities) [91]. The personal server application may also run on a PC in home monitoring settings. Each new generation of wireless handheld devices includes more processing power, storage, and battery life, thus allowing them to meet the requirements of mHealth services.

Some of the benefits of a mobile computing device in mHealth include:

1. Patients are able to receive the care they require regardless of where they are located, in addition to having increased access to their own medical information.
2. It makes it possible to simply coordinate consultations between different providers located in different parts of the world. It enables clinicians to communicate about a patient and assists in obtaining the necessary therapy for them, particularly in situations where a patient requires the care of a specialist who is not available in their area.
3. There are a variety of EHRs and/or billing systems that can make things simpler for physicians, billers, and coders by utilizing mobile technology.
4. It also has the potential to improve accuracy all throughout an organization. When using paper documents, it is difficult to maintain an orderly structure, and it is difficult to identify faults that have been made. Errors are caught by digitized systems, and when those systems are made available on mobile devices, accuracy increases at every step of the process.
5. It enables hospitals and medical practices that are more efficient. Better outcomes are typically the result of improved communication between healthcare practitioners and patients, decreased rates of medical errors, and increased ease of access for patients.

### 6.4. Communication Technologies for mHealth

**Figure 12:**
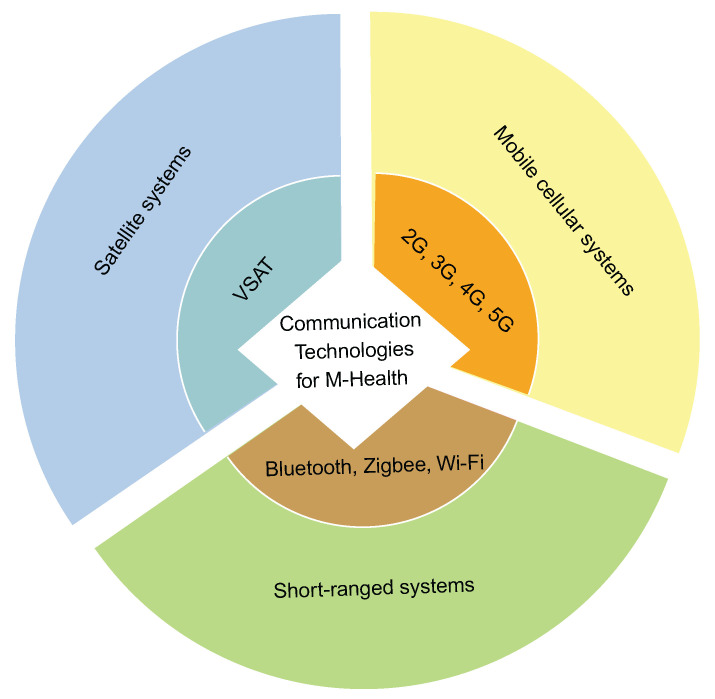
Different communication technologies for mHealth purposes.

The following is a list of the most important categories of communication technologies that can be used for mHealth-related purposes: short-ranged systems, satellite technology, and mobile cellular systems, as summarized in Figure 12. These various technologies are discussed below:

#### 6.4.1. Short-Ranged Technologies

Integrating emerging wireless solutions into eHealth has become a requirement for accurate and efficient healthcare delivery; however, it poses significant challenges in terms of interoperability, performance, and security [119]. Ultra-wideband networks (UWB), Bluetooth networks, and wireless local area networks (WLANs) are all examples of short-range wireless transmission systems (WLANs). WLANs are classified into several types, including legacy 802.11b, 802.11a, 802.11g, 802.11n, 802.11ac wave1, 802.11ac wave2, and 802.11ax networks, with maximum data transmission rates of 2, 11, 54, 54, and 450, 866.7 Mbps, 1.73 Gbps, and 2.4 Gbps, respectively [120]. The carrier frequency of 802.11, 802.11b, and 802.11g networks is 2.4 GHz, while it is 5 GHz for 802.11a and 802.11n networks.

According to [119], various low cost broadband wireless solutions have emerged over the course of the past few decades as a result of the proliferation of radio frequency (RF) and microwave techniques. These solutions include wireless personal area networks (WPANs), wireless local area networks (WLANs), cellular systems, wireless wide area networks (WWANs), and wireless metropolitan area networks (WMANs). Infrared Data Association (IrDA), Radio Frequency Identification (RFID), Bluetooth, ZigBee, and Ultra wideband are some of the other short-range technologies that have also been developed.

Following these advancements, many works have examined short-range communication technologies for use in eHealth applications. For instance, in [121], an energy harvesting-based Wi-Fi system was proposed for use in eHealth applications. In [122], Wi-Fi was used as the communication technology in an IoT-based health monitoring system. There has been the general use of WLAN as the communication network for metropolitan eHealth applications [123]. The authors in [124] designed a tele-health smart sensor device to aid home care personnel based on the Bluetooth technology. Additionally, Bluetooth was used to transmit data in a smart eHealth application for telediagnosis [125]. All of these efforts have led to the conclusion that the potential for the use of short-range technologies in eHealth is promising. A detailed comparison of the different characteristics of some short-range technologies can be found in [126,127].

#### 6.4.2. Satellite Communication for mHealth

Satellite networks have the potential to deliver eHealth services to locations that do not have access to broadband network services or any other kind of telecommunication network [128]. It has been suggested and researched to use satellite communication for eHealth in emergency situations. In particular, as a result of advancements in satellite communication technologies, satellite IP network services employing very small aperture terminals (VSATs) have been used primarily in rural areas for telemedicine [129,130].

Furthermore, a hybrid network communication system employing VSAT and wireless fidelity (Wi-Fi, IEEE802.11b/g wireless LAN) was proposed in [128] and analyzed to establish temporal network connections, particularly after a disaster. A field-tested version of this technology is already available on the market. However, despite these advancements, we mention a few of the advantages and disadvantages of satellite communications for mHealth purposes as follows:

**Advantages:**

Satellite communication technology has the following advantages [23]:

1. Comprehensive geographic coverage, along with the integration of isolated terrestrial networks (“islands”).
2. It is capable of providing Demand Assignment Multiple Access (DAMA), also known as bandwidth on demand.
3. It serves as an optional replacement for damaged fiber-optic networks for disaster recovery.
4. The Internet and satellites’ broadcasting capabilities promote multipoint-to-multipoint communications.

**Disadvantages:**

1. It has asymmetric transmission rates [128]. Even in advanced services utilizing a dish with a diameter of 1.2 m, the uplink speed is less than 2 Mbps, while the downlink speed can reach 60 Mbps. Uplink speeds are crucial for the provision of telemedicine, as high-quality still or live video images of patients must be transmitted to hospitals using uplink communication. These images are crucial for doctors to diagnose the medical conditions of victims.
2. Secondly, VSAT equipment items are frequently cumbersome [131]. When a large-scale natural catastrophe happens, it may be hard to transport VSAT equipment to some devastated areas due to severely damaged roads and halted traffic.
3. Large latency resulting from the distance traversed by data over VSAT systems, which makes them inefficient for eHealth emergency services.

There are also recent advancements in satellite technologies that have the potential to improve the delivery of eHealth services, and we explore a few of them as follows:

1. Significant advancements have been made in the signal capacity of geostationary satellites (GEOs), which can currently receive and broadcast at hundreds of gigabits per second [132]. Modern GEO satellites also operate for longer periods of time and with higher power than ever before. They will undoubtedly play a significant role in the provision of broadband Internet to developing nations with lower average earnings. This has the potential to significantly improve the delivery of eHealth services in low-income contexts.
2. Developments in Ka-band Systems for Mobile Satellite Communications (KASYMOSA) intends to expand the mobile space’s use of satellite technology. This requires advancements in antenna array, new location strategies, and a major increase in bits-per-second attributes [133]. In this regard, engineers believe that further Ka-band development will enable consumer-level satellite phone calls, enhanced emergency response efforts, and other revolutionary functions [134].
3. In recent years, there has been a significant effort to build satellite phased array antennas for broadband communications [135]. This will provide broadband capabilities to both land and marine vehicles, as well as boost worldwide Internet connectivity, which will ultimately enhance the delivery of eHealth services [136].
4. Advances in software-defined satellites will enable satellites to carry digital payloads with the essential components to transmit and receive data at ranges unimaginable a decade ago, regardless of environmental impediments such as canopy forests or mountains [137]. This innovation would eliminate the requirement for prelaunch testing, saving considerable time and money and enhancing the delivery of eHealth services.

#### 6.4.3. Mobile Cellular Systems for mHealth

The development of mobile cellular technologies has advanced significantly from the first generation to the current fifth generation (5G) and beyond. This section provides a brief overview, focusing on advancements in mHealth services as they progress from third (3G) to current (5G) technologies.

##### 3G Systems

3G mobile technologies represent a significant upgrade from the previous 2.5G communication systems. They support faster data rates and improved Quality of Service (QoS) compared to earlier technologies, thus making them usable for healthcare applications. Although wireless technologies have evolved dramatically from 3G systems to the more advanced 4G and 5G systems, nevertheless, 3G systems are still applicable for mHealth purposes particularly in low income or developing countries with low 4- and 5G penetration services. It should be mentioned that 3G systems are capable of supporting a number of healthcare services within practical bit rates ranging from 144 Kbps in mobile scenarios to 2 Mbps in indoor environments [138]. Communication technologies that are based on 3G have been deployed in many health-related applications, notably those that rely on data collecting activities and quick integration into medical records. For instance, Holter monitors have been utilized for the purpose of ECG and EEG monitoring [139].

One of the most important characteristics of 3G wireless technologies is their ability to combine previously used cellular protocols such as CDMA, GSM, and TDMA. This is accomplished through the use of the three air interface modes, namely: CDMA, CDMA2000, and the Universal Wireless Communication (UWC-136) interfaces, all of which are part of the wideband CDMA (W-CDMA) brand. Because W-CDMA is generally backward compatible with 2G GSM networks and offers a bandwidth of 5 to 10 MHz, it makes a great platform for higher-capacity applications. Additionally, it is interoperable with the TDMA (IS-36) and IS-95 networks that are already in use.

In terms of 3G-based medical application systems, the authors of [140] proposed a mobile telemedicine system. Their system serves as a platform for data collection from a variety of medical devices. Furthermore, it enables the smooth transfer and distribution of data to healthcare professionals via cellular networks powered by 3G technology. It is possible to apply this technology in any part of the world that has access to 3G networks, which makes it a significant potential for reducing mortality and morbidity while also generating financial savings. A teletrauma system that is capable of transmitting speech, video, and medical data in real time between an ambulance and a level-1 trauma hospital was introduced in [141]. This system can be helpful for pre-hospital trauma care, particularly in scenarios that involve extended transportation periods or a great amount of data transfers. It is possible that such a system could enhance the quality of trauma treatment by hastening the evaluation and management of injured patients, hence improving the likelihood of prompt and efficient intervention. Indeed, there are many other 3G-based mHealth applications, which can be found in [142,143].

Generally, a 3G system has the following advantages and disadvantages as it may relate to mHealth applications:

**Advantages:**

1. In addition to providing access to the Internet from any location, multimedia services are also accessible.
2. It offers interoperability across service providers as well as inexpensive call rates all around the world.
3. Customers are able to make use of wireless broadband, and it is able to support applications that are fairly data-intensive.
4. It is a lot faster than the previous networks.

**Disadvantages:**

1. It is possible that it will not supply sufficient bandwidth due to the data-intensive requirements of mHealth applications.
2. It has a high power consumption rate overall.
3. It requires base stations to be densely deployed in order to achieve quicker rates, and as a result, medical applications that use this technology may be expensive to deploy.
4. Roaming, data/voice services, and integration of these features for health applications have not yet been fully implemented.

##### 4G Systems

Fourth generation (4G) broadband cellular network technologies are the successor to 3G and the forerunner to 5G systems. WIMAX technology based on IEEE802.16 standards and the Third Generation Partnership Project (3GPP) Long Term Evolution (LTE) are the two broad candidates for 4G systems [144]. They are both required to offer the capabilities outlined by the International Telecommunication Union (ITU) in IMT Advanced systems [145]. Some of these requirements include being an all-IP packet switching network with peak download data throughputs of at least 1 Gbps (under low mobility) and 100 Mbps (under high mobility). However, neither WIMAX nor LTE currently support these throughputs; however, they provide far faster rates than 3G systems [146]. The technical parameters of 4G networks have been extensively covered in the literature, with a few important sources for the interested reader in [147,148,149].

4G systems have been widely deployed for various mHealth applications. A number of studies have been published in the field of 4G-health systems. For instance, the authors in [150] evaluated the medical quality of service (QoS) and quality of experience (QoE) for 4G-health systems. In this instance, the word “4G-health” refers to the long-term evolution of mHealth, which can be defined as the progression of mHealth toward targeted personalized medical systems that have adaptable features and are compatible with 4G networks [151]. 4G has also been used in applications that have to do with medication optimization [152], for remote patient monitoring [153], innovative assistive technologies [154], and diabetes management systems [155], to name a few of these applications.

The following are highlights of some of the technological advantages and downsides of 4G, with a view toward its use in applications related to medical care:

**Advantages:**

1. In comparison to 3G systems, it offers superior spectral efficiency as well as increased speed, capacity, and bandwidth.
2. It is possible to attain a higher level of network security, which in turn makes it possible to have a high level of usability: at any time, anywhere, and with any type of technology. This provides support for multimedia services at a low transmission cost, which is very much needed in mHealth applications.
3. It is able to achieve lower cost per bit, in addition to providing a seamless network of multiple protocols and air interfaces, which ultimately results in a communication system that is substantially more cost-effective.
4. It increases the level of synchronization among different devices, which in turn provides worldwide access, service portability, and a variety of service quality options, all of which will enhance mHealth application capabilities.

**Disadvantages:**

1. There are not currently many regions that have 4G connectivity, which may restrict its use for mobile health applications on a wider scale.
2. Protocols and standardization for networks used in medical applications have not yet been determined.
3. The rate at which it consumes power is still fairly high, which means that it will be disadvantageous to sensors that are used in medical applications and are limited by battery life.

##### 5G Systems

5G is a newly emerging technology that differs from 3G and 4G in that it provides advanced processing capabilities as well as a virtualized computing platform to enable mobile IoT (mIoT) [156]. The number of connected devices that can be supported by 5G, the speed of the network, and exceptionally low latency are the characteristics that set it apart from its predecessors. Several innovations have significantly increased the performance of 5G systems, including the provision of an end-to-end network architecture, network slicing, and virtual evolved packet core (vEPC) for switching and data processing [157]. 5G networks, like previous generations, are made up of several cell sites with sectors that send coded signals. Each cell site is linked to the main network backbone via fast wireless or wired backhaul. The 5G network, like 4G LTE, uses OFDM encoding, but more efficiently to provide faster speeds. However, the air interface for 5G is designed to be more flexible and have lower latency than LTE [157]. These developments have generated promising prospects for enhancing mHealth services.

Thus, there have been significant developments in mHealth applications due to 5G technology. For instance, a new 5G eHealth architecture based on optical camera communication (OCC) was presented in [158]. Here, the authors proposed an OCC system to gather data from wearable sensors for monitoring purposes. These OCC systems were then linked to 5G access networks in order to communicate with a main network. They demonstrated that the proposed system can accomplish rapid and secure connection for monitoring numerous patients simultaneously. In [159], a comparable architecture and protocol for smart, continuous eHealth monitoring via 5G was presented. Essentially, these and other architectures utilize 5G as the enabling communication technology to realize real time transfer of health data in order to significantly improve medical service delivery. The notion of 5G slicing was also employed to develop an innovative eHealth system in [160]. Their solution was designed to collect heterogeneous medical data from a range of 5G-connected medical devices. Several survey publications have also focused on evaluating eHealth applications over 5G networks, such as in [18,161,162].

Nevertheless, the 5G technology is not without flaws; therefore, we will highlight a few noteworthy benefits and drawbacks that may have an impact on the delivery of eHealth services, as follows:

**Advantages:**

1. It possesses a very high speed (>1 Gbps in high mobility), much higher capacity than previous technologies, increased efficiency, and is optimized for longer battery lifetime.
2. It has a lower cost per bit, supports multimedia, voice, and Internet services, and enables the delivery of images at significantly higher resolutions.
3. It provides large bidirectional bandwidth for mobile users, the highest possible quality of service (QoS), and the ability to deliver uniform, continuous, and consistent connectivity across the entire world.
4. In addition to providing worldwide access and service portability as well as support for a variety of service types, it intends to provide a technology that brings together all similar 5G networks on a single platform.

**Disadvantages:**

1. Due to the fact that it is a relatively new technology, the cost of its development infrastructure is expensive.
2. There are still some privacy and security concerns that have not been fully addressed, such as the prevention of eavesdropping, and these concerns need to be addressed for flawless eHealth service delivery.
3. It will be a costly endeavor because many of the older devices (1G, 2G, 3G, and 4G) will not be compatible with the 5G standard and will need to be replaced with brand-new models.
4. This technology is still in the process of being developed, and investigations into its practicality are now taking place.

We have also provided a quick qualitative comparison in Table 5 of the major communication technologies based on their bandwidth, coverage, and mobility to better highlight the information provided in this section.

### 6.5. Summary of Discussion

This section has examined trends in mHealth within three important areas: mobile sensors, mobile computing devices, and communication technologies, and the following take-aways and recommendations are noted:

#### 6.5.1. Our Take-Aways

1. The development of mobile sensors has seen significant progress in recent years, which has led to improvements in the accuracy with which medical parameters may be measured. The widespread adoption of more advanced smartphones, which come equipped with in-built sensors such as accelerometers and gyroscopes, is largely responsible for the recent advances made in sensor technologies.
2. On the basis of the progress made in computing devices, there are many new and improved mobile phones, PDAs, and portable media devices that can process information faster. These gadgets have increased the potential for not just measuring health data, but also processing and analyzing them and providing feedback to patients.
3. In terms of advancements in communication technologies, 5G technology stands out as the future technology for the delivery of eHealth services. Because of its ability to provide higher peak data transmission speeds of multiple gigabits per second (Gbps), extremely low latency, and increased reliability, 5G wireless technology will have a substantial impact on the enhancement of mHealth services and applications. Additionally, it will deliver huge network capacity, increased availability, and a more consistent user experience to a larger number of people.

#### 6.5.2. Our Recommendations

1. While much has been published about the development of wireless biosensors for use in wireless body area networks and general eHealth systems, enhanced mobile computing devices based on new smart phone technologies, and communication technologies pertaining to 5G systems, there is still a great deal to be covered in the literature. For instance, further miniaturization of biosensors within the scope of nanotechnologies, and standardized frameworks for the risk assessment of the use of mHealth applications in eHealth systems are areas that may require more research and future synthesis of existing literature.
2. Despite the fact that 5G networks have the potential to improve the reach and functionality of eHealth systems, their coverage regions remain inadequate. Therefore, it is suggested that future research should concentrate on the potentials for seamless integration between 5G and satellite communications technologies in order to increase the performance of eHealth coverage.
3. Improving the degree of eHealth system awareness and utilization literacy among users is a source of concern. In this regard, there are less studies and investigations on novel literacy models for increasing mHealth acceptance among users, and it is advised that more research be conducted in this area.

## 7. eHealth: Security and Privacy Concerns

In this section, we highlight the security and privacy concerns associated with eHealth systems. Given that ubiquitous eHealth service delivery has the capacity to continually monitor the health conditions of patients, it is therefore of the utmost importance to ensure that data is secured and kept private throughout the monitoring, storage, and retrieval phases of an eHealth system. In this context, we note that significant progress has been made in the literature to strengthen wireless data security and privacy in eHealth. Thus, in this section, we simply attempt to summarize the most recent developments in this field. Therefore, we first explore the unique aim of securing eHealth systems, then we highlight the challenges or types of attacks against eHealth systems, and finally the existing solutions to these problems.

### 7.1. Goals of Securing eHealth Systems

There are three main aspects to be considered when attempting to secure any eHealth information system [163], which are as follows:

1. Confidentiality: This entails the assurance that sensitive data will not be disclosed to unauthorized elements. Such a requirement must be maintained to safeguard a patient’s anonymity [164]. However, because eHealth systems are typically either based on an edge- or cloud-designed architecture, thus when data control is handed over to a cloud service, the information in question becomes accessible to an increased number of users, which in turn increases the likelihood that the data may be compromised [165]. This challenge is presently aggravated owing to the ever increasing number of parties, devices, and applications involved in eHealth systems, which results in an increase in the number of potential threats to the confidentiality of data circulating in the system. Thus, it is imperative for a patient to have confidence that the eHealth system would maintain the confidentiality of his or her information in order for the patient–doctor interaction to be trusted. If the patient believes that the information provided to a doctor is not safeguarded and that their privacy is compromised, trust breaks down, which can ultimately ruin the essence of eHealth systems in general.
2. Integrity: This refers to the prevention of illegal changes to any component of the data within an eHealth system. It is absolutely necessary that there is never a breach in the validity of the data that is recorded or sent within an eHealth system. This is crucial to ensuring that the medical records of patients are correct and consistent with the information that was intended. Because medical records of patients are required for physicians to make diagnoses and decisions regarding treatment, any unauthorized access to those data, change or loss of such records can be immensely destructive to the whole existence and sustaining of eHealth systems in general [166]. Consequently, the HIPAA Security Rule (Section 164.312(c) (1) Integrity) [167] stipulates that public resources are required to “implement policies and procedures to protect electronic personal healthcare information from improper alteration or destruction”. Thus, before accessing the data, applications that store and handle patient information in a healthcare context are required to incorporate integrity and verification features, just as is the case with non-medical applications. This can be accomplished through the use of checksums or hashes. In the event that the integrity check is unsuccessful, the healthcare application is required to report an error and exit without processing any of the data [165].
3. Availability: This means that the service or data rendered by eHealth systems should always be available when required. The availability of eHealth systems is indeed critical because a patient’s life may be jeopardized if medical services are denied [168]. Such accessibility of data encompasses the ability to continue operations despite the misbehavior of some authorities as well as following a breach in security. Furthermore, eHealth systems should be able to minimize service interruptions caused by events such as power failures, failed hardware, system upgrades, and denial-of-service assaults. In addition, such systems should be able to maintain the usability of medical records after HIPAA security and privacy regulations have been enforced.

Other issues listed in [165], such as ownership and privacy of healthcare information, nonrepudiation, and access control anonymity, fall under the assurance of confidentiality, whereas authenticity, auditing, unlinkability, and secured transmission are subsets of integrity.

### 7.2. Security Threats against eHealth Systems

A comprehensive list of security attacks and vulnerabilities is difficult to compile because new types of attacks are invented on a regular basis, and some of them cannot be conceived of until the attack has been carried out. On the other hand, it is quite possible that some of the vulnerabilities found in the systems of today, as well as those that are being studied for future ubiquitous computing systems, will also be present in eHealth systems, such as:

1. Eavesdropping: As medical information is collected, transferred, and stored across different eHealth systems, attackers could attempt to obtain access to such information. An example of this would be an unauthorized listen-in on a radio conversation that is taking place between wireless sensors, followed by the capturing of data. Because medical information is both private and highly sensitive to alternations, this must be avoided.
2. Inaccurate patient information and erroneous system actions may occur from attackers being able to edit medical data while they are being gathered, sent, or stored. This can happen when attackers are able to alter the information when it is being acquired, sent, or stored. This may result in false alarms, such as the activation of alerts, and may lead, for example, to rescue operations that are not essential. Even worse, false negatives, in which worrisome data are changed to produce normal results, can conceal abnormal or emergency situations.
3. Similar to the preceding point, attackers are able to forge alarms on medical data by simply creating phony messages rather than changing legitimate ones. Forgery of alarms on medical data is a common form of cybercrime. This can once again result in inaccurate data recordings or a bogus system.
4. Denial of Service: When a system is jammed or overloaded, it becomes inoperable. In the worst-case scenario, sick or injured people will be unable to receive the necessary care, which can lead to fatalities. In [169], the impacts of denial of service on the routing of data in mobile eHealth networks were examined. Different solutions to the problem of denial of service are often accompanied by additional constraints related to the use of cryptography, such as the difficulty in authenticating routing packets. Therefore, denial of service remains a problem that must be resolved in order to improve the performance of eHealth systems.
5. User location tracking: Because eHealth system users leave continuous records of messages sent out, and because the system also might expressly support person localization, this data might be gathered, consolidated, and analyzed to obtain very detailed location profiles. This is certainly an invasion of privacy that must be avoided. Nonetheless, as noted by [170], token-based employee mobility monitoring remains a widespread technique of staff management today. Employees who do not use or possess the device may be refused access to certain places. Such transaction-logging techniques also permit mobility tracking, retrospective evaluation of movements, and maybe even real time prediction capabilities relating to the person’s likely destination. The surveillance of people’s locations is generating concern among privacy activists [171]. The terms “Uberveillance” and “dataveillance” (a combination of data and surveillance) have both become buzz terms in the fight against overwatching (surveillance) systems [172]. Such movements may create a challenge for eHealth systems as it will be argued that sensors can be hijacked and used to invade the privacy of individuals for malicious purposes.
6. User activity tracking: This form of threat is unique to eHealth systems. It may be feasible to examine people’s activities based on the data collected. When an individual is constantly monitored, it may be able to determine their activities merely by examining their heartrate and oxygen concentration data [173]. Such medical monitoring is a feature of ReMoteCare, as explained in [174]. Insurance providers could use this knowledge to deny benefits to persons who live an unhealthy lifestyle. This, once again, has an impact on a user’s privacy. Furthermore, smart phones with GPS modules can enable network operators to conduct a location estimate within minutes after receiving a police inquiry [175]. Furthermore, location intelligence can expose a considerable measure about one’s tastes, acquaintances, relationships, and behaviors [175].
7. Physical manipulation: Because it may not be particularly difficult to gain access to the wireless sensors, particular those attached to equipment, attackers may try to steal equipment, tamper with the sensor devices in order to change the sensor values, or just throw them away, or damage them.

### 7.3. Security Solutions for eHealth Systems

Effective security measures implemented in eHealth systems are deployed either during the data collection, transmission, and/or storage phases. We explore existing solutions under these three categories as follows:

#### 7.3.1. Safe Data Collection

In eHealth systems, sensors are necessary to collect medical data either in isolated or networked enviroments, such as within wireless body area networks (WBANs) or as single implanted sensors, aimed at recording data streams such as pulse rate, blood pressure, ECG, and EEG, to mention a few. These data are often immediately or later transferred to a common processing unit such as a home server or mobile device. In this regard, a few methods such as the use of elliptic curve cryptography (ECC) and/or other hybrid encryption algorithms have been developed and adopted because they enable security with reduced key sizes between wireless sensor nodes and connecting devices [176,177].

Incorporating a central authenticating module in an eHealth system is another example of a decentralized strategy [178,179]. This approach is used to validate patients, physicians, and clients who are actively using the network. The actors are required to identify themselves to this authority and provide evidence in order to receive authorization. Patients, physicians, and other stakeholders are all directed to the central authority, which acts as the primary distribution point for cloud-based eHealth servers. Such systems can adopt crypto-based solutions such that when using cryptosystems with symmetric keys, session keys are kept around for the duration of several transaction timestamps [180]. The central authority will be in possession of the master key, which enables secure communication to take place between the personal computers used by patients and the cloud server. As a result of this configuration, individuals have ownership over their health information by way of a trusted authority that serves as an intermediary to maintain and secure their health information from commercial healthcare services, or any other unauthorized entity. This enables individuals to make more informed decisions about their care. A reliable authority thus verifies the integrity of the access controls just before the data is distributed to the other components of the setup.

There are additional noteworthy initiatives at securing data collection, specifically mitigating against jamming attacks or sensor node capture threats. For example, in [181], the authors stated that there are often two types of sensors deployed in an eHealth system: sensors put on or in a patient’s body and those deployed in hospital premises/embedded in some equipment, such as a smart hospital bed. In essence, such sensors can be compromised by an adversary, their cryptographic keys and state information can be acquired, and they can be cloned to install numerous malicious sensors and compromise the entire system. As solutions, symmetric key cryptography (SKC) and public key cryptography (PKC) have been utilized extensively. Furthermore in [181], the authors provided a summary of a variety of such systems, including the use of Blundo’s key pre-distribution technique for establishing a role-based access control (RBAC) protocol [182]. In this solution, polynomial keys are pre-distributed to patients so that they can effortlessly build pair wise keys with any authorized party and encrypt their data using these keys.

#### 7.3.2. Data Transmission/Retransmission Security

Multiple kinds of cyberattacks are aimed at the transmission and retransmission of sensed data and electronic health records. Controlling who can access medical information and encrypting their contents are two efficient ways to safeguard these data. Eavesdropping, denial of service attacks, and data modification are the most significant types of attacks that can be launched in this scenario. In this context, we discuss a few potential solutions.

In [183], a privacy scheme against global eavesdropping (SAGE) for eHealth systems was proposed. The proposed solution was noted as capable of achieving both content-based and contextual privacy against known adversaries. Under a robust global adversary model, formal security proofs demonstrated that SAGE can ensure not only content-oriented privacy but also contextual privacy. In addition, the system’s performance was shown to improve transmission delay efficiency. A patient-centric access control scheme for eHealth systems, termed PEACE, was proposed in [184]. This scheme was described as being both effective and secure. The authors developed distinct access privileges for data requesters based on the responsibilities of the data requesters, and they then assigned distinct attribute sets to each of the data requesters. By implementing digital signature and pseudo-identity approaches, the PEACE system was able to ensure that the integrity and confidentiality of personal health information were maintained. Extensive investigations into both the scheme’s performance and its security showed that the PEACE protocol was able to meet the needed level of security while only incurring an acceptable communication delay. Similar to PEACE, an enabling security and patient-centric access control (ESPAC) for eHealth in cloud computing was proposed in [185] by the same authors. Essentially, there was little to no difference between the proposed schemes (i.e., PEACE and ESPAC).

Other solutions for securing data transmission in eHealth systems have recently emerged, such as one based on the use of authenticated keys, such as the improved fast and secure CAMEL-based authenticated key in smart health care systems proposed in [186]. Another solution is based on a remote secured system using European Telecommunications Standards Institute (ETSI) SmartBAN as an enabling technology [187], and an intriguing Deoxyribonucleic Acid (DNA)-based ECC solution for an end-to-end secured scheme in [188]. In particular, the DNA-based ECC technique in [188] leverages DNA sequences based on biological processes to encrypt and decrypt medical data. As a novel technique, it was tested and found to outperform existing methods against relevant threat models. Machine learning approaches, such as in [189], are also beginning to come to the fore against security attacks in eHealth networks. For instance, in [189], the authors developed a differentially private federated learning method for anomaly detection in eHealth networks. In this case, network traffic was protected using a federated learning-based jointly trained anomaly detection system. This enabled real time traffic anomaly identification while maintaining hospital collaboration and keeping local data secure and private.

#### 7.3.3. Safe Data Storage

There are security and privacy problems wherever medical data are stored and processed, i.e., in EHR servers as well as on the personal computers of health care providers. Several specifications, such as the German eHC and standardizations including the HL7 and ISO/TC 215, have made significant efforts to protect the security of medical data by implementing access control mechanisms and encrypting data [190].

Storing sensitive information in centralized data centers raises the possibility of data leakage to unauthorized parties. Sensitive data must be adequately protected, for example, by the use of strong cryptographic encryption. Administrators must also be able to run and maintain the data center without access to patient data.

Another issue that is rarely addressed is the security of end-user systems. The PCs and network equipment at the doctor’s office, as well as the computer platforms of hospital information systems, are examples of end-user systems. Medical doctors, in particular, who run their own small practice, rarely have the expertise or time to professionally manage their IT systems in order to safeguard them against malware attacks [191]. They use their computer systems, on the other hand, not only to access their patients’ health records, but also for other applications such as billing systems and Internet browsers. However, today’s commodity operating systems lack advanced security features and are not built as robustly as high-assurance systems. Due to architectural constraints, they do not provide enough runtime protection for programs and operating system software, and they lack information flow management techniques and secure user interfaces [192]. All of this makes these systems vulnerable to virus attacks, which might steal passwords and secret data or leak sensitive information to unapproved Internet destinations. Furthermore, those computer systems are generally used by a group of individuals, such as medical assistants, who may connect them to mobile storage devices, such as USB memory sticks, to convey data to other platforms. Typically, data transferred in this manner escapes the control of any eHealth infrastructure security safeguards.

There have been a number of recent advancements made in the quest to solve the issue of secure data storage in eHealth systems. For example, in [193], a modified blowfish algorithm was utilized in order to keep patient data safe and protected when stored on a variety of different systems. The authors stated that it takes their technique 72% and 48% reduction in time to encrypt and decrypt files. The authors were able to accomplish this by increasing the block size of the encryption platform used from 64 bits to 124 bits. In addition to this, they asserted that their method is one of the quickest square codes that are being used at the moment, making it suitable for use on a large variety of processors. In order to encrypt medical data, the authors in [194] developed the Paillier cryptosystem and a digital signature. This allowed them to send medical data with access privacy settings when the data were transferred using wireless sensors. They used a common entity model in a domain information model (DIM), which was responsible for the administration and control of medical devices.

Markov models were used in [195] when attempting to describe a stationary stochastic mechanism. The IoMT-based personal condition monitoring system was proposed in their system because it may not restrict human movement. In a different proposition in [196], the adaptability and versatility of Ethereum Blockchain was leveraged, which led to its use as the platform of choice in their solution. Consequently, users’ data can then be stored within the same file system on a network of storage nodes which are distributed utilizing the design of an interplanetary filing system. This idea thus removes the requirement for a centralized server. As a possible component of the master key management technique, many modules, including key generation and encryption, have been suggested.

When it comes to obtaining speedier access to healthcare content, using keys from a multi-key registry solution is often advised [197]. The indexes of multiple sources that are encrypted and merged makes it possible for a cloud server to integrate numerous data encryption indices from different health organizations with a specific customer without putting the patient’s right to privacy in jeopardy [198]. The confidentiality of involved parties and their data privacy are protected when there is an effective data-sharing strategy in place. Thus, the Tate pairing and the Weil pairing are two common examples of bilinear pairing functions. Both of these transformations are linear representations of cyclic groups, which have been used for data security purposes [199].

Concerns over the confidentiality and safety of patient information may, on the other hand, prompt some medical facilities to implement EMRs in a more measured fashion than others. RBAC is the preferable access control architecture, with passwords/logins and digital signatures as the optimal authentication techniques in electronic health record systems [37]. This conclusion was reached after a significant amount of work was conducted on the subject. As the state of information technology has continued to improve, there has been mounting pressure placed on healthcare organizations to make the switch from manual to electronic systems. Electronic health records are quickly becoming one of the most important information technology systems, and they have caught the attention of both public and private medical institutions. However, the implementation of electronic health records has been shown to be a challenging undertaking, particularly in countries that are not yet considered developed. Therefore, after conducting in-depth research on the topic, a comprehensive study came to the conclusion that electronic health records (EHR) had a sizeable positive impact on the quality of healthcare by enhancing the health of patients and ensuring treatment that is effective, efficient, well timed, unbiased, and patient-centered [200].

### 7.4. Summary of Discussion

This section has discussed security in eHealth systems from a number of different perspectives. These perspectives include first being aware of what the goals of any security solution should be, then being aware of the various types of attacks and threats that may arise against eHealth systems, and lastly being aware of the recent solutions to these problems. Our take-aways and recommendations from this section are as follows:

#### 7.4.1. Our Take-Aways

1. The goal of securing eHealth systems is broadly covered in terms of guaranteeing confidentiality, integrity, and availability. Other goals fall under these areas such as ownership and privacy of healthcare information, nonrepudiation, and access control anonymity, falling under confidentiality, whereas authenticity, auditing, unlinkability, and secured transmission falling under integrity.
2. Due to the sensitive nature of the eHealth industry, it is crucial that all of the threats such as denial of service, eavesdropping, and data falsification, are adequately addressed, with some security solutions mentioned in Section 7.2.
3. In a nutshell, methods relating to the use of cryptographic encryption techniques, solutions based on blockchain technology, heuristic algorithms, and the physical protection of end nodes have been well documented in the literature.

#### 7.4.2. Our Recommendations

1. It is evident that there is no one solution that can address all of the concerns regarding the security of eHealth systems. As a result, data should be protected either at the site of generation, i.e., at the sensors themselves, or during transmission or retransmission, and ultimately, during storage or access of the data. This should be the focus of both current and future solutions.
2. There are further understudied areas, such as enhanced consensus methodologies for strengthening blockchain solutions, the creation of privacy-enforced eHealth systems, and optimizing key management complications to decrease communication overheads. As a result of the lack of study on these and many other topics, this section cannot be considered exhaustive. Nonetheless, in its present state, this section has provided some minimum context and synthesized information to developers who are either involved in developing or implementing secured eHealth systems towards improving the performance of such systems.

## 8. eHealth: Research Challenges and Future Directions

The purpose of this section is to highlight a few of the most critical research challenges, as well as to present our opinions on potential and future solutions. In spite of the progress made in the development of eHealth systems, a wide variety of research challenges remain unresolved. However, we must note that it is almost impossible to identify all of these research challenges that need to be resolved in order to develop eHealth systems that are both viable and efficient. This is because the study and development of eHealth systems has blossomed into a broad area of research. Nevertheless, in order to give a coherent overview of these issues and prospective research directions, we have structured this section as follows: first, we highlight the research challenges and future direction associated with eHealth architectures. The discussion of the research issues and future direction in mHealth and security follows a similar format. This makes it easier for readers to immediately access particular sections on challenges and future direction, as opposed to combining the two conversations.

### 8.1. Research Challenges in Architectures

- Standardization: eHealth is currently undergoing a period of rapid transition on multiple fronts, including the economic, social, and technical levels. Standardization is an innately delicate subject, and the fact that there are many initiatives taking place in a variety of countries only adds to the complexity. There are already established norms and frameworks in the field of security, but little to none within the unifying the architectural domain. Consequently, there is need for research in this regard to avoid compatibility issues. In terms of research, it is still unclear what the proper procedure needs to be in order to guarantee compatibility of technologies, consistency of manufacturing, and objectivity of measurement. This difficulty may be connected to the fact that different tasks are required at different times of the lifespan of standards, which further affects the various players. In order to accomplish this goal, some sort of systematic and conceptual framework will be required; hence, more research activities will be necessary. For the purpose of carrying out a survey that is more accurate regarding the present status and the desired state of the field, improved data collection procedures will also be necessary. This will make it possible to enhance standardization processes. In addition, the methods of standardization that will be necessary to achieve uniformity in the endorsement and description of healthcare systems, the structure of information, types of data, semantic consistency, and the handling of electronic patient records are not yet known. This is another area in which there is a lack of knowledge. These significant research obstacles need to be overcome in order to make progress toward the development of unified architectures for eHealth systems.
- Weak interdisciplinary collaborations: There is a lack of synergy between the engineers and IT specialists who are in charge of creating eHealth technologies and the health experts who are responsible for producing the theoretical contents. This frequently results in poorly constructed designs that do not fulfill all of the requirements posed by the people utilizing the health services. For instance, those who specialize in technology may create websites that are engaging and entertaining but are not founded on any health behavior theory, whereas those who specialize in health education may create programs that are grounded in theory but do not make full use of the potential offered by technology to bring the subject matter to life [201]. In terms of research, it remains to be determined which strategy for message tailoring will be most effective for enhancing inter-disciplinary collaborations. In this instance, tailored (i.e., customized) messaging generates individualized communication with a higher likelihood of conveying convincing health messages to target groups. Other research obstacles include establishing the optimal method for delivering eHealth interventions that foster stronger partnerships and result in enhanced eHealth content. In addition, the construction of objective metrics and machine learning algorithms for evaluating and finding the most relevant specialists better suited to multidisciplinary collaborations remains an untapped field of research that deserves further study.
- Need for higher communication speed: High-speed transmission of data from some source (such as a patient or record server) to a destination (a medical practicioner or administrator) will be essential to the success of any and all prospective eHealth designs. Consequently, the adoption of eHealth systems and the provision of services will require enhanced data rates that go beyond the capabilities of the currently available 5G technology. Specifically, it is well known that existing 5G technologies cannot completely support eHealth systems, particularly those that require ultra-reliable low latency communications (URLLC). According to [161], even though retransmission and grant-free transmission (GFT) can improve communication reliability, it will also result in increased transmission delay. Therefore, further study will be necessary in the field of data speed optimization in order to overcome these concerns. Other areas of research required to increase transmission speed include the improvement of medical data fusion and mining algorithms, as well as compression techniques.
- Latency between edge–cloud interactions: It is common knowledge that using cloud services will result in delays, particularly delays caused by the communication channel or the processing power of the cloud server. Consequently, such latencies can be extremely detrimental to the provision of eHealth services, particularly in circumstances involving an emergency. Such limitations can lead to non-availability of cloud services, which can be further detrimental to eHealth service delivery. In terms of research, it is necessary to develop self-organizing networks that can automatically and rapidly adjust to congested links in order to improve transmission performance. To control service outages that may result from edge–cloud interactions, improved software and hardware installation, updates, and reconfiguration methods must be established. These are areas of unknown knowledge, which will require further studies. In addition, this will also demand specialized eHealth platforms to enhance service delivery, necessitating more study into the development of improved data capture algorithms and aggregation techniques to identify novel medical patterns for heterogeneous data sourcing and transmission.

### 8.2. Future Directions in eHealth Architectures

- When designing and developing eHealth systems and architectures, it is essential to utilize multidisciplinary teams and formative research. Consequently, academic boards, as well as commercial and governmental organizations that award grants, need to devise and implement rules that demand a diverse group of researchers and developers with expertise across the several fields that are relevant to the health business.
- New architectures should be developed based on the use of better technologies. An example of this can be seen in [158] where 5G technology was combined with optical camera communication (OCC) to develop a reliable and low-latency architecture for eHealth solutions.
- Architectures that are highly distributed, component-based, and self-organizing should be the target of future research and development activities. In this context, a variety of different ideas, such as edge intelligence, machine learning techniques, and quantum computing technologies, can be utilized to great effect.

### 8.3. Research Challenges in mHealth

- Low levels of health literacy: It is of the utmost importance to determine how mHealth applications are created, developed, and supplied in order to guarantee that such applications are comprehensible to all people and can be acted on by them. This is significant since the effectiveness of the intervention of mHealth applications can only be achieved for users with a high level of health literacy, but users with a low level of health literacy are frequently disregarded, which is something that continues to be a challenge. As a result, mobile health applications need to be designed following methodologies that are considered industry standard in order to provide information in a manner that is accessible to the various audiences they are aimed at. Research-wise, the optimal framework for defining eHealth literacy demands and barriers toward producing new solutions remains unknown, thus warranting future studies. Furthermore, improved methodologies for literacy type profiling will be necessary to generate a more comprehensive list of resources to benefit health practitioners in promoting literacy improvement. Lastly, further research will be required in developing new eHealth literacy models that will enhance existing models such as the Lily model toward providing additional dimensions that will improve eHealth literacy [202].
- The price of smartphones and miniaturization of sensors: Even though the price of smartphones may be coming down as a result of advancements in technology, the reality is that not everyone can afford such devices. As a result, this can be a barrier for those unfortunate people who do not have the financial means to purchase a smartphone. Therefore, the ability to supply specialized low cost devices for the delivery of eHealth services should be considered a subject of urgent study by both developers and policy managers. In this case, a great deal of research must be undertaken in order to enhance access to eHealth services through the miniaturization of biosensors. Efforts to improve the sensitivity of point-of-care (PoC) diagnostics, for instance, have become crucial for the early diagnosis of illnesses. In this instance, it is vital to investigate how new materials might be created to enable the re-use of microfluidic systems in order to build cheaper and more ecologically friendly technologies [203]. The synergistic integration of optics and microfluids to allow new capabilities without sacrificing integrability or compactness is another area of study that has the potential to lower costs and warrants further investigation. This kind of research into microfluids and optics can lead to better microfluidic drug delivery and screening methods that are less dangerous and work better.
- Lack of frameworks for conducting risk assessments of mHealth applications: The fast growth of mHealth applications makes it necessary for government health agencies to pay attention to legislation regarding the risk assessment of mHealth apps. Unfortunately, these current and future frameworks have not yet been defined, especially with regards to the use of mobile auditing systems that permit real time monitoring of mHealth apps. This raises research problems in identifying how mHealth apps consume resources and the capacity to warn users if abnormal resource usage patterns are discovered. In this context, more research is required to create algorithms that allow the recording of fetal cardiac events onto a mobile phone for subsequent processing and evaluation of fetal risk [204]. How to design methods and procedures for identifying harmful mHealth apps, where the accuracy of mHealth apps can be simply evaluated and determined, continues to be investigated. In addition, it is crucial to conduct research on how to combine multiple usage situations, contextual variables, and program complexity in order to estimate the total probability and degree of harm caused by mHealth apps. By finding answers in these areas of research, it will be possible to make and use eHealth applications that are safer.
- Poor or lack of communication network coverage: Once more, all mHealth applications are dependent on communication technologies, particularly wireless solutions. On the other hand, there are regions that are not covered or places that are difficult to access, such as rural areas or the outskirts of towns and cities. When this transpires, mHealth and eHealth services, in general, will be rendered completely inoperable. An area of research interest is the need for ways to offset such occurrences or inadequately covered regions. In this context, further studies on long-distance communication technologies will be necessary. In this case, this may involve investigations and studies into the fields of satellite and low-power long-range communications for the delivery of eHealth services. Particularly intriguing will be research into the improvement of miniature satellites (also known as CubeSats) for eHealth applications. This will need tackling obstacles such as how to create ways for effectively integrating CubeSats with other communication technologies, such as geostationary and medium Earth orbit satellites and 5G technologies, as well as how to optimize the amount of onboard transceivers in CubeSats to enhance data scheduling. These and other intriguing research gaps, such as the development of software-defined networking solutions for broadband satellite communications, should also be investigated in the future to improve the delivery of eHealth services.

### 8.4. Future Directions in mHealth

- It is recommended that more medical research be carried out in order to create sensor-based technologies that may be utilized in the process of monitoring patients, carrying out patient diagnoses, and administering treatments. These have the potential to make use of cutting-edge sensor technologies in the area of nanoscience and nanotechnology.
- The development of home monitoring systems that include affordable terminals for caregivers and patients, in addition to a central monitoring system, is an area for future consideration. Such centralized hubs may take the form of web-based systems that hold a central database, or they may even be mist or edge computing solutions. These concepts may assist to reduce the cost of purchasing medical devices as well as increase the quality of service that is provided by eHealth systems.
- The requirement for specialized working groups has become necessary in order to hasten the pace at which breakthroughs in mHealth are being made. In fields such as wireless body area networks (WBANs), work groups such as the IEEE 802.15.6 have been established for low-power wearable devices or body implants. These groups are intended for use in the human body. Additionally, such organizations are required in the field of mHealth in order to better specialize their efforts.
- Long-range communication: The potentials of long-range communications may be utilized in the provision of eHealth services to hard-to-reach locations such as rural areas or the outskirts of towns. These kinds of technologies include LoRa, SigFox, NB-IoT, and a variety of other types of long-range communication systems. However, before these technologies can be put to use, a number of research issues, including efficient management of energy and high data rates, need to be resolved. This may be a topic for more investigation in the future.

### 8.5. Research Challenges in eHealth Security

#### 8.5.1. Challenges with Blockchain Technology

The following are a few of the most important concerns that are associated with the implementation of blockchain technology in eHealth systems:

- Lack of awareness: In the first place, there is a lack of awareness of how the technology may need to function in the eHealth sector, as well as a lack of awareness of the technology itself, particularly in industries other than banking. This, in turn, may make it more difficult to invest in and investigate the viability of this concept across eHealth systems.
- Scalability: The capacity to handle a large number of users simultaneously is still a concern for the blockchain business. The processing of a single transaction using blockchain technology requires the use of several complicated algorithms. Because of the potentially enormous number of users, which often spans both national and international boundaries, this will be a significant obstacle for its implementation inside eHealth systems. In particular, it will be required to investigate how to optimize the huge block sizes, which are frequently the cause of the slow propagation speed in blockchain technologies. This will entail research activities aimed at resolving the blockchain problem, i.e., blockchain storage optimization. On the other hand, it may necessitate a complete redesign of the blockchain, which may necessitate new knowledge on how to decouple traditional blocks into smaller, efficient blocks with improved building techniques.
- Confidentiality: Despite the fact that blockchain is suitable as a solution to security concerns in eHealth systems, blockchain remains an open ledger that is viewable by everyone. This makes it possible for anybody to verify the accuracy of the blockchain. This has the potential to become a liability, especially in delicate contexts like those seen in eHealth system setups. In this situation, more research may be necessary to address privacy leakage concerns, which may include the development of information regarding how to use mixing services. The use of mixing services offers anonymity by adopting many input addresses to multiple output addresses, making it challenging to determine the origins of information. In addition, further study is necessary to create zero-knowledge proof. In this scenario, blockchain miners would not be needed to validate a transaction using a digital signature, but would instead validate currencies against a list of valid coins [205]. It is largely uncertain how such an approach would function, thus necessitating additional research and investigation.
- Cost: Blockchain is still in its infancy as a technology, and despite the fact that it could be useful in eHealth systems, there is still a significant amount of work to be done in the field of study about this topic. Consequently, it will be challenging to integrate it into existing systems, necessitating the development of whole new systems, which will result in substantial additional expenses. Because of this, its adoption by participants in the private or even public health industries may be hampered.

#### 8.5.2. Challenges with Cloud Services

The move toward cloud-based environments in eHealth raises concerns about privacy, security, access control, and compliance. This is due to the inherent security challenges that are associated with cloud technology. Despite the appealing features that cloud services provide, by storing personal health records on public cloud servers, patients lose their ability to exercise direct physical control over their data, which poses a potential risk to patient confidentiality. Thus, when it comes to storing and accessing data in the cloud, one of the most challenging issues has been the question of data security and data integrity. This has led to further research questions, noted as follows:

- How can fool-proof security measures be enabled in cloud services?
- How can enforced privacy be achieved in eHealth systems?
- Are there better access control mechanisms for the secured transfer of EHRs?
- How can health data can be effectively shared among multiple healthcare providers?
- How can insider attacks be minimized, particularly involving administrative staff with accessibility options?
- How can key management complexity be handled while sharing healthcare data between disparate healthcare providers?

#### 8.5.3. Other General Challenges with eHealth Security

The HITECH Act and its corresponding regulations present a number of research problems, some of which are highlighted here, with further details available in [206]:

- The need for dependable user and entity authentication methods and systems, which must be capable of achieving the goals of unique identity, multi-factor authentication, and role-based authorization.
- A lack of robust access control measures that are particularly stringent, such as those that offer emergency access protocols, automated logouts (i.e., system time outs), device control lists, physical access restrictions, and enforced policies for system and data access control.
- Inadequate audit control systems with robust capabilities for logging and monitoring activities.
- There is need for enhanced data and record transmission security mechanisms over wireless communication channels.
- A lack of an integrated solutions that include the deployment of administrative, technological, and physical safeguard mechanisms, as well as security training and awareness of such tools as being part of compliance preparation for the uptake of eHealth systems.

### 8.6. Future Directions in eHealth Security

- There may be a need to accelerate policies and blueprints for research in blockchain for digital health and patient uptake. This could include initiating government-led digital education and adoption campaigns about the technology. This may improve awareness about the technology and enable smoother application integration to prevent information overload and confusion among health care workers (HCWs) and patients concerning the technology.
- Certificates, like any other public key infrastructure, must be managed to assure the authenticity of key holders (smartcards, connections, servers, and so on). This involves certificate issuance and distribution, as well as updating revocation lists.
- Other components, in addition to the cryptographic infrastructure, must be handled and maintained. This comprises the hardware and software components used in EHR servers, billing servers, and health care provider computer devices. Smartcard readers and connectors to secured networks, for example, should be properly approved and tested. A secure distribution system is required for the installation and updating of software components. On the one hand, valid software upgrades must be able to cause changes in program configuration. Unauthorized and malicious alterations, on the other hand, must be detectable in order to stop future usage or to exclude infected components from the eHealth infrastructure.
- A permitted patient-centric blockchain for EHRs that overcomes most of the present obstacles in the cloud might be a future solution and path for dealing with eHealth privacy problems.
- A variety of systems have used the role-based access control (RBAC) approach to protect eHealth security and privacy. We believe that future study should look at the use of the attribute-based access control (ABAC) architecture, which may offer superior scalability and flexibility for authentications and authorizations. This remains an opinion and could probably be an area for future research.
- Attribute-based encryption (ABE) is also acknowledged to be effective at protecting privacy in eHealth; nevertheless, excessive calculations during decryption of data are impending, thereby impacting its efficiency. We also believe that finding a solution to these bi-linear procedures would improve ABE’s efficiency. The hunt for a solution is seen as a promising eHealth topic of interest.
- General enforcement of privacy standards (termed enforced privacy) should be implemented. The majority of the proposed solutions concentrated their efforts primarily on protecting the patients’ confidentiality and safety. However, all parties participating in eHealth systems should have their privacy enforced. Thus, devising the right mechanisms in this regard will be a useful future direction in eHealth studies.

## 9. Conclusions

This article has presented a detailed survey of three facets of eHealth: architecture, mHealth, and security concerns. We have discussed blockchain-, IoT-, and cloud-based architectures as contemporary examples of notable designs for eHealth systems. Our conclusion is that the development of a unified architecture will necessitate the merging of various distinct designs, each of which has its own advantages and disadvantages. In addition, we examined mHealth deployment in eHealth with an emphasis on mobile computing devices and wireless sensors in eHealth, as well as mHealth communication technologies used in eHealth with reference to short-ranged, satellite, and mobile cellular systems. We are convinced that the development of 5G networks and subsequent generations will be extremely important to improving mHealth service delivery in eHealth systems. The concept of security and privacy in eHealth was also examined, with an emphasis on the objectives of a secure system, the actual threats, and the existing solutions to these problems. Again, we reach the conclusion that there may be no single method for ensuring the security of all eHealth systems. Instead, different solutions are required at the source (sensor) level, during transmission/retransmission of data or records, and during the storage and access stages. Finally, we remark that there are several challenges that must be surmounted in order to develop and improve the viability and effectiveness of eHealth systems, many of which have been highlighted in the last section of the article. This survey article has thus focused on three essential topics, but it cannot claim to be exhaustive due to the vast amount of research still required to implement successful eHealth systems. Therefore, future works can build on the research challenges and plausible directions outlined in this article, such as issues pertaining to the standardization of eHealth architectures, the development of a unified architecture, and the improvement of blockchain technologies to enhance security performance. Nevertheless, the future of eHealth systems seems promising and practical, but this may be contingent on the need for further research and investment from both the private and public sectors of the health industry.

## Figures and Tables

**Table 1:** Summary of the related work.

Title	Year	Category	Specific Details
[23]	2003	QoS	Latest technologies in wireless communication Existing mobile Technology Wireless technologies in eHealth
[4]	2006	Country	eHealth in Austria Solutions to interoperability using ODP standards eHealth in specific country (Austria)
[20]	2009	QoS	QoS in Wireless eHealth, Handoff Schemes for QoS QoS in eHealth
[9]	2011	Cloud	Components of cloud computing Cloud computing as related to eHealth Cloud computing in eHealth
[24]	2012	IoT	eHealth Monitoring (EHM) ecosystem Current EHM market segments Monitoring Systems in eHealth
[6]	2012	IoT/M2M	Applications of M2M communications to eHealth M2M standards for eHealth M2M in eHealth
[10]	2012	Cloud	An overview on “eHealth cloud” Deals with eHealth cloud opportunities Addresses eHealth cloud challenges Contribution to thebuilding of eHealth cloud Cloud and Security in eHealth
[5]	2014	Country	Current eHealth systems in public and private sectors Challenges facing eHealth in Bangladesh eHealth in Bangladesh
[21]	2014	QoS	Key features of present-day eHealth applications QoS in term of specific medical care service QoS in wireless network QoS in eHealth
[25]	2014	Security	Deals with privacy approaches in eHealth Need and requirement for privacy in eHealth Privacy approaches in eHealth and comparism Cloud and security in eHealth
[22]	2015	QoS	User attitudes Extracting Knowledge from social media Social Network
[15]	2015	Security	Explores the security and privacy issues in eHealth Comprehensive overview of biometric technology in addressing eHealth security challenges Security challenges
[7]	2016	Standardization	Relevant standards for eHealth Infrastructure Infrastructure challenges and limitations of Radioaccess technology in eHealth Standardization of e health infrastructure
[6]	2016	Standardization	Key challenges of M2M M2M standards for eHealth Technology for IoT devices Standardization of M2M communication
[11]	2017	IoT	IoT in medical environment and market places Problems regarding response time and precision Explore IT architectures able to ensure security andprivacy Wearable and energy saving properties IoT devices in medical environment
[26]	2017	Standardization	EHRs Databases Interoperability in maintain EHRs EHR Standardization
[18]	2019	IoT	5G technology in wireless Body Area Network (WBAN) Explored the pros and cons of WBAN healthcare system Architecture of WBAN based on 5G technology The roles of mm wave in communication Application of 5G technology in WBAN
[19]	2019	Security	Threats in Bluetooth communication for eHealth systems Examples of attacks performed on eHealth systems IoT devices
[16]	2019	Security	Analysis and security of medical data Health Data Issues Healthcare data privacy Security and privacy of medical data
[14]	2019	Security	Security and Privacy requirements of eHealth datain cloud Security and privacy of medical data Cloud issue in eHealth
[17]	2020	IoT	The utilization of intelligent techniques in health The integration of IoT devices and cloud computing Security and privacy of medical data in IoT

**Table 2:** Comparison between blockchain and IoT/cloud services.

Characteristics	Blockchain	Cloud Services
Data ownership	Cryptographic keys and Algorithm	Central Authority
Privacy and Security	Cryptographic Authentication	Central Authority
Access Control	Inherently Identical for all permissioned nodes	Central Authority
Trust	Native via Immutable records	Established via Central Authority
Stored Procedures	Smart contracts	Not Available
Transaction creation	Available to all permissioned parties	Managed via Central authority

**Table 3:** Other types of architectures deployed in eHealth purposes.

Architectures	Purpose
WEBRTC [80]	Remote examination of patients and injured persons in case of accident
IoT GDPR controller [81]	Gives data owner a full control of his data, setting security policies, modifying them on run time, tracking data flow and notification
IoT Continua [82]	Ensure the remote management of personal health devices and gateways
Vicinity [83]	Combine AAL (Ambient Assisted Living) and mHealth to provide functionalities such as authentication, authorization and end-to-end encryption
Fuzzy-based HAR [84]	An IoT-based architecture used to continuously acquire data from body sensors
Insole optical fibre sensor [85]	Adaptable to shoe sole for plantar monitoring. Monitors the foot plantar pressure distribution during gait (Walking movement)
Scalable eHealth [86]	Clinic hardware communication with other clinic hardware remotely without human interaction

**Table 4:** Different wireless sensors used in mHealth.

Sensors	Functions
ECG (Electrocardiograph)	Monitors heart activity, cardiac arrhythmias, heart failure [104]
EMG (Electromyography)	Monitors muscle activity [105] & Ergonomics [106]
EEG (Electroencephalography)	Monitors brain electrical activity [107] & Stress recognition [108]
PPG (Photoplethysmograph)	Monitors pulse and blood oxygen saturation [109] & monitor hypopnea (i.e., sleep condition) [110]
Cuff-based pressure sensor	Monitors blood pressure [111]
Resistive or Piezo Elelctric chest bell sensor	Monitors respiration & Respiratory tract infections [112]
Tilt sensor	Monitoring trunk position [113]
Gyroscope Sensor	Gait-phase detection [114]
Accelerometer	Estimates type and level of users activity [115]
Smart sock/insole sensor	Count steps/fall detection [116]

**Table 5:** Summary of communication technologies deployable for mHealth applications.

Technology	Bandwidth	Coverage	Mobility
2G/2.5G (GSM/GPRS)	Low	Rural/Suburban/Urban	Low
3G (UMTS)	Average	Suburban/Urban	Average
3.5G (Wimax, LTE)	High	Suburban/Urban	Average
4G (Wimax-ver2 LTE Advance)	High	Rural/Suburban/Urban	High
5G	Very high	Rural/suburban/Urban	Very high
VANET	High	Urban	High
Zigbee	Low	Rural/suburban/Urban	Low
Bluetooth	Low	Rural/Suburban/Urban	Low
Wi-Fi	High	Suburban/Urban	Low

## Data Availability

Not applicable.
